# ADNP Controls Gene Expression Through Local Chromatin Architecture by Association With BRG1 and CHD4

**DOI:** 10.3389/fcell.2020.00553

**Published:** 2020-07-01

**Authors:** XiaoYun Sun, WenJun Yu, Li Li, YuHua Sun

**Affiliations:** ^1^The Key Laboratory of Aquatic Biodiversity and Conservation, Institute of Hydrobiology, Chinese Academy of Sciences, Wuhan, China; ^2^Hubei Key Laboratory of Agricultural Bioinformatics, College of Informatics, Huazhong Agricultural University, Wuhan, China; ^3^The Innovation of Seed Design, Chinese Academy of Sciences, Wuhan, China

**Keywords:** ADNP, embryonic stem cells, chromatin, lineage-specifying genes, BRG1, CHD4

## Abstract

ADNP (Activity Dependent Neuroprotective Protein) is proposed as a neuroprotective protein whose aberrant expression has been frequently linked to rare neural developmental disorders and cancers, including the recently described neurodevelopmental Helsmoortel-Van der Aa syndrome. Recent studies have suggested that ADNP functions as an important chromatin regulator. However, how ADNP-regulated chromatin mechanisms control gene expression and stem cell fate commitment remains unclear. Here we show that ADNP interacts with two chromatin remodelers, BRG1 and CHD4. ADNP is required for proper establishment of chromatin accessibility, nucleosome configuration, and bivalent histone modifications of developmental genes. Loss of ADNP leads to enhancer over-activation and increased ratio of H3K4me3/H3K27me3 at key primitive endoderm (PrE) gene promoters, resulting in prominent up-regulation of these genes and priming ES cell differentiation toward endodermal cell types. Thus, our work revealed a key role of ADNP in the establishment of local chromatin landscape and structure of developmental genes by association with BRG1 and CHD4. These findings provide further insights into the role of ADNP in the pathology of the Helsmoortel-Van der Aa syndrome.

## Introduction

Embryonic stem cells (ESCs) possess an epigenome and chromatin structures that are required for the maintenance of self-renewal and pluripotency. The ES-specific chromatin state is directly or indirectly regulated by various factors, including epigenetic regulators and signaling molecules (Gifford et al., 2013). Chromatin remodelers are epigenetic regulators that use ATPase activity for nucleosome assembly and organization, chromatin access and nucleosome editing (Chen and Dent, 2014; Clapier et al., 2017). Great progress has been made in understanding the biochemical composition of the chromatin remodeler complexes and their role in ES cell self-renewal and pluripotency has been firmly established (Ho et al., 2008; Kidder et al., 2009; Lu and Roberts, 2013; Zhang et al., 2014; O’Shaughnessy-Kirwan et al., 2015; Zhao et al., 2017).

Activity Dependent Neuroprotective Protein was first described as a neuroprotective protein and has been implicated in various rare neural developmental disorders and cancers, including the Helsmoortel-Van der Aa syndrome, gastric and colorectal cancers (Pinhasov et al., 2003; Vandeweyer et al., 2014). The Helsmoortel-Van der Aa syndrome is characterized by global developmental delay, intellectual disability, dysmorphic features, hypotonia and autism (Helsmoortel et al., 2014). However, the molecular mechanism underlying the syndrome remains poorly understood. ADNP contains nine zinc fingers and a homeobox domain, suggesting that it functions as a transcription factor. Consistently, ADNP deficiency in pluripotent P19 cells leads to aberrant gene activity, functioning as both transcriptional activator and repressor (Gozes et al., 2015). A growing body of research has shown that ADNP functions as an important chromatin regulator by physical association with chromatin remodelers. For instance, ADNP was shown to interact with core sub-units of the SWI/SNF chromatin remodeling complex such as BRG1 and BAF250 (Mandel and Gozes, 2007). By association with the chromatin regulator HP1, ADNP localizes to pericentromeric heterochromatin regions where it silences major satellite repeat elements (Mosch et al., 2011). ADNP forms a stable tripartite complex with CHD4 and HP1 (called the ChAHP) to control lineage gene expression in ESCs (Ostapcuk et al., 2018). Recently, it has been shown that ADNP regulates local chromatin architecture by competing for binding with CTCF, a master genome architecture protein (Phillips-Cremins et al., 2013; Kaaij et al., 2019).

Although approximately 15,000 ADNP bound sites were identified in ESCs, most ADNP ChIP-seq peaks are not localized at gene promoters (Ostapcuk et al., 2018). In addition, many genes bound by ADNP are not deregulated in the absence of ADNP (this work and Kaaij et al., 2019). Thus, the mechanism by which ADNP regulates gene expression remains unclear. In ES cells, most developmental transcription factors are in bivalent state which is characterized by the presence of both H3K4me3 and H3K27me3 at gene promoters. The bivalent domains are proposed to silence developmental genes in ES cells while keeping them poised for later activation (Ku et al., 2008). The enhancers of developmental genes are usually in a “poised” state, premarked by H3K4me1/H3K27me3; while the enhancers of pluripotency-related genes are marked by H3K27ac, a mark associated with active enhancers (Calo and Wysocka, 2013).

In this work, we hypothesize that the ADNP-regulated chromatin-remodeling mechanism contributes to ES cell gene expression state by modulating bivalent histone modifications and chromatin accessibility. We show that ADNP functions as a key chromatin regulator- this is potentially linked to its interaction with the chromatin remodelers, BRG1 and CHD4. ADNP is required for proper establishment of local chromatin accessibility, nucleosome configuration, and bivalent modifications of developmental genes. Loss of ADNP leads to enhancer over-activation and increased ratio of H3K4me3/H3K27me3 at key PrE gene promoters, resulting in prominent up-regulation of these genes and priming ES cell differentiation toward endodermal cell types. These findings provide further insights into the role of ADNP in the maintenance of ES cell phenotype and the pathology of the Helsmoortel-Van der Aa syndrome.

## Materials and Methods

### ES Cell Culture

Mouse embryonic stem cells (mESCs) R1 were maintained in Dulbecco’s Modified Eagle Medium (DMEM, BI, 01-052-1ACS) high glucose media containing 10% fetal bovine serum (FBS, Gibco, 10099141), 10% knockout serum replacement (KSR, Gibco, 10828028), 1 mM sodium pyruvate (Sigma, S8636), 2 mM L-Glutamine (Sigma, G7513), 1,000 U/ml leukemia inhibitory factor (LIF, Millipore, ESG1107) and penicillin/streptomycin (Gibco, 15140-122) at 37°C with 5% CO2.

The 2i culture condition was used as described previously (Chappell et al., 2013). The commercial ESGRO-2i Medium (Merck-Millipore, SF-016-200) was also used when necessary. We found that in 2i medium, *Adnp-/-* ESCs adopted morphology indistinguishable to that of control ESCs, and maintain self-renewal capacity for more than 20 passages that we tested.

### Embryoid Body (EB) Formation

Embryonic stem cells differentiation into embryoid bodies was performed in attachment or suspension culture in medium lacking LIF or knockout serum replacement (KSR), as described in our previous report (Chappell et al., 2013).

### *Adnp* shRNA Knockdown

The shRNA plasmids for *Adnp* (TRCN0000081670; TRCN0000081671), and the *gfp* control (RHS4459) were purchased from Dharmacon (United States). To make lentivirus, shRNA plasmids and *Trans*-lenti shRNA packaging plasmids were co-transfected into H293T cells according to the kit manual (Open Biosystems, TLP4615). After determining the virus titer, mESCs were transduced at a multiplicity of infection of 5:1. Puromycin selection (1 μg/ml) was applied for 4 days to select cells with stable viral integration. Quantitative PCR (qPCR) and Western blot were used to assess the knockdown of *Adnp*.

### Generation of *Adnp-/-* ESCs

*Adnp-/-* mESCs were generated by CRISPR/Cas9 technology. Briefly, we designed two sgRNAs on exon 4 of the *Adnp* gene by using the online website http://crispr.mit.edu/. The sgRNAs sequences are: sgRNA1: 5′-CCCTTCTCTTACGAAAAATCAGG-3′; sgRNA2: 5′-CTACTTGGTGCGCTGGAGTTTGG-3′. SgRNAs were cloned into the pUC57-U6 expression vector with G418 resistance. The plasmids containing sgRNA and hCas9 were co-transfected into mESCs using Lipofectamine 2000 (Gibco). After 48 h, mESCs were selected with 500 μg/ml G418 for 7 days. Then the cells were re-seeded on 10 cm dishes coated with 0.1% gelatin to form colonies. The single colony was picked up and trypsinized for passage. DNA from the passaged cells was extracted and used for genotyping. At least two mutant ES cell lines were established in the lab.

### Generation of 3 **×** FLAG Tagged *Adnp-/-* mESC Cell Lines

The full-length *Adnp* cDNA (NM_009628.3) was amplified by PCR and then cloned into pCMV-3 × Flag vector. The full-length *Adnp* cDNA sequence containing N-terminal 3 × Flag sequence was subcloned into the pCAG-IRES-Puro vector. To make stable transgenic cells, *Adnp-/-* mESCs were transfected with pCAG-IRES-Puro-3 × FLAG-Adnp vector using Lipofectamine 2000 (Gibco). 48 h later, cells were selected by 1 μg/ml puromycin. After 4–5 days drug selection, cells were expanded and passaged. Western blot assays were performed to confirm the transgenic cell line using FLAG antibodies.

Inducible transgenic cell lines were established according to the manual of the Tet-Express inducible expression systems (Clontech, 631169). Briefly, *Adnp-/-* ESCs were transfected with 2 μg pTRE3G-3 × FLAG-*Adnp* with linear 100 ng puromycin marker using Lipofectamine 2000 transfection reagent. 96 h later, 1 μg/ml puromycin was added and drug selection was performed for 2 weeks to establish the stable transgenic cell line. To induce target gene expression, 3 × 10^6^ transgenic cells were plated in 6-well plates. The next day, the Tet-Express transactivator (Clontech, 631178) was added (3 μl Tet-Express to a final 100 μl total volume according to the kit manual) for 1 h in serum-free medium to induce target gene expression. Then cells were allowed to grow in complete medium for an additional 12–24 h before assaying for target protein induction. Western blotting was used to assess target protein expression levels using FLAG antibodies. In the absence of Tet-Express transactivator, pTRE3G provides very low background expression, whereas addition of Tet-Express proteins strongly transactivates target genes.

### RNA Preparation, RT-qPCR and RNA-Seq

Total RNA from mESCs was extracted with a Total RNA isolation kit (Omega, United States). 1 μg RNA was reverse transcribed into cDNA with TransScript All-in-One First-Strand cDNA synthesis Supermix (TransGen Biotech, China). Quantitative real-time PCR (RT-qPCR) was performed on a Bio-Rad qPCR instrument using Hieff qPCR SYBR Green Master Mix (Yeasen, China). The primers used for RT-qPCR are listed in Tables 2, 3. All experiments were repeated for three times. The relative gene expression levels were calculated based on the 2^–ΔΔCt^ method. Data are shown as means ± S.D. The Student’s *t* test was used for the statistical analysis. The significance is indicated as follows: ^∗^*p* < 0.05; ^∗∗^*p* < 0.01; ^∗∗∗^*p* < 0.001.

For RNA-Seq, mESCs were collected and treated with Trizol for RNA extraction. The isolated RNAs were quantified by a NanoDrop instrument, and sent to BGI Shenzhen (Wuhan, China) for whole RNA-Seq libraries and deep sequencing. RNA-Seq experiments were repeated for three times. Differentially expressed genes (DEGs) were defined by FDR < 0.05 and a Log_2_ fold change > 1 was deemed to be DEGs.

### Protein Extraction, and Western Blot Analysis

For protein extraction, ES cells and EBs were harvested and lysed in TEN buffer (50 mM Tris–HCl, 150 mM NaCl, 5 mM EDTA, 1% Triton X-100, 0.5% Na-Deoxycholate, with Roche cOmplete Protease Inhibitor). The lysates were quantified by the Bradford method and used for Western blot assay. Antibodies used for WB were ADNP (R&D Systems, AF5919, 1:500), FLAG (F3165, Sigma, 1:1000), HA (66006-1-Ig, Proteintech, 1:1000), BRG1 (21634-1-AP, Proteintech, 1:1000), CHD4 (ab181370, Abcam, 1:1000), SOX17 (24903-1-AP, Proteintech, 1:1000), GATA4 (19530-1-AP, Proteintech, 1:1000) and GATA6 (55435-1-AP, Proteintech, 1:1000). WB assay was performed as described previously. Briefly, the proteins were separated by 10% SDS-PAGE and transferred to a PVDF membrane. After blocking with 5% (w/v) non-fat milk for 1 h at room temperature, the membrane was incubated overnight at 4°C with the primary antibodies. Then the membranes were incubated with a HRP-conjugated goat anti-rabbit IgG (GtxRb-003-DHRPX, ImmunoReagents, 1:5000), a HRP-linked anti-mouse IgG (7076S, Cell Signaling Technology, 1:5000) for 1 h at room temperature. The GE ImageQuant LAS4000 mini luminescent image analyzer was used for photography. Western blots were repeated at least two times. Quantification of WB band intensity was performed by use of ImageJ software.

### Co-immunoprecipitation (Co-IP)

Co-immunoprecipitation was performed for either ESCs or HEK293T cells as described in the text. Before performing co-IP, stable or transgenic cell lines were established as described above. For making transgenic cells, the full length or partial cDNAs of *Chd4* (geneID: 107932), *Brg1* (*Smarca4*, geneID: 20586) and *Adnp* genes were amplified by PCR and then cloned into the pCAG vector. The primers used for PCR are listed in Table 1. The constructs were verified by DNA sequencing. Co-IP experiments were performed with Dynabeads Protein G (Life Technologies, United States) according to the manufacturer’s instructions. Briefly, 1.5 mg Dynabeads was conjugated with 10 μg IgG, or 10 μg anti-ADNP antibody, or 10 μg anti-FLAG antibody, or 10 μg anti-HA antibody, or 10 μg anti-BRG1 antibody, or 10 μg anti-CHD4 antibody. The whole cell lysates from cells were incubated with antibody-coupled Dynabeads overnight at 4°C. The next day, the beads were washed with PBST and boiled with loading buffer for 5 min. The protein samples were run on a SDS-PAGE gel and transferred to a PVDF membrane. The membrane was blocked with 5% (w/v) non-fat milk for 1 h at room temperature (RT), and followed overnight at 4°C with antibodies against ADNP (R&D Systems, AF5919, 1:500), FLAG (F3165, Sigma, 1:1000), HA (66006-1-Ig, Proteintech, 1:1000), BRG1 (21634-1-AP, Proteintech, 1:1000), CHD4 (ab181370, Abcam, 1:1000). Next day, the membranes were incubated with secondary antibodies (HRP-conjugated goat anti-rabbit IgG (GtxRb-003-DHRPX, ImmunoReagents, 1:5000), or HRP-linked anti-mouse IgG (7076S, Cell Signaling Technology, 1:5000) for 1 h at room temperature. After three times wash with PBST, the ECL substrate (Pierce, #32109) was applied for detection of signals. The GE ImageQuant LAS4000 mini luminescent image analyzer was used for photography.

**TABLE 1:** The primers for qRT-qPCR.

**Mouse genes**	**Forward (5′-3**′)	**Reverse (5**′**-3**′)
*β-actin*	AGAGGGAAATCGTGCGTGAC	CAATAGTGATGACCTGGCCGT
*Nanog*	ACCCAACTTGGAACAACCAG	CGTAAGGCTGCAGAAAGTCC
*Pou5f1*	CGTTCTCTTTGGAAAGGTGTTC	GAACCATACTCGAACCACATCC
*Pax6*	AGTGAATGGGCGGAGTTATG	ACTTGGACGGGAACTGACAC
*Nestin*	CCCTGAAGTCGAGGAGCTG	CCCTGAAGTCGAGGAGCTG
*Gsc*	GCACCATCTTCACCGATGAG	AGGAGGATCGCTTCTGTCGT
*Brachyury/T*	CTGGGAGCTCAGTTCTTTCG	CCCCTTCATACATCGGAGAA
*Gata4*	TCTCACTATGGGCACAGCAG	GCGATGTCTGAGTGACAGGA
*Gata6*	CAAAAGCTTGCTCCGGTAAC	TGAGGTGGTCGCTTGTGTAG
*Sox17*	GCTTCTCTGCCAAGGTCAAC	CTCGGGGATGTAAAGGTGAA

**TABLE 2:** The primers for ChIP-qPCR.

**Mouse genes**	**Forward (5**′**-3**′)	**Reverse (5**′**-3**′)
*Park2 P1*	CTGGGATCCGAGGCTAGAGT	ACCAGCGTTTCTGTCAGGTT
*Sox17 P2*	ACTAGTCTTGGGAAAGCGCC	AGAAAGAAAGCCCGGGGATG
*Gata4 P1*	CTAACGGGCCTGGTGTTCTT	CCCACTCACAGGGTGACTTC
*Gata6 P1*	TTTAGGGCTCGGTGAGTCCA	GAGGAAACAACCGAACCTCG
*Nanog P1*	CATCACGTCGGACTGCTTCT	CAGGGTTTCTCGTCCTTTCCT
*pou5f1 P2*	TGGAGACTTTGCAGCCTGAG	TTCTAGTCCACACTGCGTCG
*Pax6 P1*	ACGACGAAAGAGAGGATGCC	GGGCTTTCGCTGGAAGTAGA
*Sox1 P1*	GGCTGAGCTGAGTGCAAAGT	GGGTCGTGTTTAAATGCGCT

**TABLE 3:** The primers for plasmid constructions.

**Mouse genes**	**Forward (5**′**-3**′)	**Reverse (5**′**-3**′)
*Adnp*	ATGTTCCAACTTCCTGT CAACAATC	GCATATGGGCCGT GTTGCATC
*Adnp-N*	ATGTTCCAACTTCCTG TCAACAATC	TCACAATGTCAAA TCAAAGCTCAAAG
*Adnp-C*	ATGGTTCATATTGATG AAGAGATGG	GCATATGGGCCGTGT TGCATC
*Adnp* (1–735)	ATGTTCCAACT TCCTGTCAACAATC	TCATTCATGGTCCTC AATGACATGCT
*Adnp* (733–1473)	ATGGAACGGATA GGCTATCAGGTC	TCAGAGGCATTTG CTAGTAAAATTGTG
*Adnp* (1450–2055)	ATGCACAATTTTA CTAGCAAATGCCTC	TCAGTGGACTAG ATGCAGAGTGAT
*Adnp* (2035–2451)	ATGATCACTCTGC ATCTAGTCCAC	TCAGTACTTTTC ACAGTCGCGGAC
*Adnp* (2430–3371)	ATGGTCCGCGACT GTGAAAAGTAC	GCATATGGGC CGTGTTGCATC

Mapping experiments were performed in HEK293T cells. 2 × 10^7^ cells were seeded in 10 cm dishes without antibiotics in DMEM medium containing 10% FBS at 37°C with 5% CO2. 24 h later, the plasmids containing a gene of interest were transfected into HEK293T cells using Lipofectamine 2000 (Gibco) according to the manufacturer’s instructions. And 48 h later, the cells were harvested for the co-IP experiments.

### Sequential Immunoprecipitation

3 × Flag-Tagged-ADNP Adnp-/- mESCs were seeded in 10 cm dishes and allowed to grow to 80–90% confluence. The cells were treated with 10 μM MG132 for 3 h and then harvested with a cell scraper. The lysate was prepared with lysis buffer containing 50 mM Tris–HCl (PH7.4), 150 mM NaCl, 1 mM EDTA, 1% Triton X-100 and 1 × ROCHE protease inhibitor. Sequential IP was carried out as follows: 1.5 mg Dynabeads was conjugated with 10 μg anti-FLAG antibody (F3165, Sigma) at room temperature for 2 h, then the lysates were incubated with antibody coupled Dynabeads overnight at 4°C with rotation. After washing with IP wash buffer (50 mM Tris–HCl PH 7.4 and 150 mM NaCl) 3 times, 0.5 mg/mL 3 × FLAG peptides (F4799, Sigma) was added and incubated with the washed Dynabeads overnight at 4°C with rotation. Next day, the supernatants were collected by a magnetic stand and used for second round IP. 50 μl supernatants were saved as input. The remainder of the supernatants were incubated with the Dynabeads pre-coupled with anti-CHD4 antibody (21634-1-AP, Proteintech) overnight at 4°C with rotation. After extensive washes, the Dynabeads were resuspended with 5 × loading buffer. Then the mixture was boiled at 95°C for 5 min, followed by Western blot assay using the anti-BRG1 antibody.

### Chromatin Immunoprecipitation (ChIP) and ChIP-seq

Chromatin Immunoprecipitation experiments were performed according to the Agilent Mammalian ChIP-on-chip manual as described (Singh et al., 2015). Briefly, 1 × 10^8^ ES cells were fixed with 1% formaldehyde for 10 min at room temperature. Then the reactions were stopped by 0.125 M Glycine for 5 min with rotating. The fixed chromatin were sonicated to an average of 200–500 bp (for ChIP-Seq) or 500–1,000 bp (for ChIP-qPCR) using the S2 Covaris Sonication System (United States) according to the manual. Then Triton X-100 was added to the sonicated chromatin solutions to a final concentration of 0.1%. After centrifugation, 50 μl of supernatants were saved as input. The remainder of the chromatin solution was incubated with Dynabeads previously coupled with 10 μg ChIP grade antibodies (ADNP, R&D Systems, AF5919; H3K4me3, Abcam, ab1012; H3K27me3, Abcam, ab192985) overnight at 4°C with rotation. Next day, after 7 times washing with the wash buffer, the complexes were reverse cross-linked overnight at 65°C. DNAs were extracted by hydroxybenzene-chloroform:isoamylalcohol and purified by a Phase Lock Gel (Tiangen, China).

For ChIP-PCR, the ChIPed DNA were dissolved in 100 μl distilled water. Quantitative real-time PCR (qRT-PCR) was performed using a Bio-Rad qPCR instrument. The enrichment was calculated relative to the amount of input as described. All experiments were repeated at least for three times. The relative gene expression levels were calculated based on the 2^–ΔΔCt^ method. Data were shown as means ± S.D. The Student’s *t* test was used for the statistical analysis. The significance is indicated as follows: ^∗^*p* < 0.05; ^∗∗^*p* < 0.01; ^∗∗∗^*p* < 0.001.

For ChIP-seq, the ChIPed DNA were dissolved in 15 μl distilled water. Library constructions and deep sequencing were done by the BGI Shenzhen (Wuhan, China). All ChIP-seq experiments were repeated two times.

### Calculation of H3K4me3/H3K27me3 Ratio at Gene Promoters

The promoter chromatin state was calculated as the relative ratio of the signal derived from the number of H3K4me3 and H3K27me3 sequence reads across a window between −3 and +3 kb of the annotated TSS. The relationship between H3K4me3/H3K27me3 ratio and expression was calculated by averaging of the H3K4me3/H3K27me3 ratio within a sliding window 100 observations wide, incrementing by 1, using a Spearman rank correlation. The ratio for *Adnp-/-* ESCs was relative to that of control ESCs. The calculation was based on the two ChIP-seq replicates. Data were shown as means ± S.D. The Student’s *t* test was used for the statistical analysis. The significance is indicated as follows: ^∗^*p* < 0.05; ^∗∗^*p* < 0.01; ^∗∗∗^*p* < 0.001.

### ATAC-seq Assay

A 50,000 control ESCs and *Adnp-/-* ESCs in LIF-KSR medium were used for ATAC-seq assay. The experiment was performed in biological replicates using two independent isogenic cell lines for each genotype. Library preparation and ATAC-seq experiments were done by the BGI company (Wuhan, China). Libraries were paired-end sequenced (2 × 75 bp) using an Illumina NextSeq 500 device.

### Immunoprecipitation in Combination With Mass Spectrometry

For IP-Mass spectrometry, the IP samples (previously immunoprecipitated by IgG or ADNP antibody) were run on SDS-PAGE gels and stained with Coomassie Brilliant Blue. Next, the entire lanes for each IP samples were cut out and transferred into a 15 ml tube containing 1 ml deionized water. Further sample treatment and the Mass Spectrometry analysis were done by the GeneCreate Biological Engineering Company (Wuhan, China).

### Immunofluorescence Assay

Cells previously seeded onto glass slides were fixed with 4% paraformaldehyde for 10 min at room temperature. Then cells were washed with ice-cold PBST three times. Following the incubation with blocking buffer (5% normal horse serum, 0.1% Triton X-100, in PBS) at room temperature for 1 h, cells were incubated with primary antibodies anti-OCT3/4 (N-19) (sc-8628, Santa Cruz) at 4°C overnight. After three-times washing with PBST, the cells were incubated with the secondary antibodies (1: 500 dilution in antibody buffer, Alexa Fluor, Thermo Fisher) at room temperature for 1 h in the dark. The nuclei were stained with DAPI (D9542, Sigma, 1:1000). After washing with PBS twice, the slides were mounted with 100% glycerol on histological slides. Images were taken by a Leica SP8 laser scanning confocal microscope (Wetzlar, Germany).

### Protein-Protein Interaction Assay Using a Rabbit Reticulocyte Lysate System

The Protein-Protein Interaction Assay using the Rabbit Reticulocyte Lysate System has been described (Sun et al., 2018). FLAG or HA tagged-ADNP, BRG1 or CHD4 proteins were synthesized using the TNT coupled reticulocyte lysate system according to the manual (L5020, Promega, United States). Briefly, 1 μg of a circular PCS2-version of plasmid DNA was added directly to the TNT lysate and incubated for 1.5 h at 30°C. 1 μl of the reaction products was subjected to WB assay to evaluate the synthesized protein. For protein-protein interaction assay, 5–10 μl of the synthesized HA or FLAG tagged proteins were mixed in a 1.5 mL tube loaded with TEN buffer, and the mixture was shaken for 30 min at room temperature. Next, IP or pull-down assay was performed using Dynabeads protein G coupled with FLAG or HA antibodies as described above.

### Alkaline Phosphatase (AP) Staining

Alkaline phosphatase activity of mESCs was performed with a Leukocyte Alkaline Phosphatase Kit (Sigma, 86C-1KT) according to the manufacturer’s instructions as described previously (Chappell et al., 2013).

## Bioinformatics Analysis

### ChIP-seq Analysis

ChIP-seq data were aligned in Bowtie2 (version 2.2.5) with default settings. Non-aligning and multiple mappers were filtered out. Peaks were called on replicates using the corresponding inputs as background. MACS2 (version 2.1.1) was run with the default parameters. Peaks detected in at least two out of three replicates were kept for further analysis. BigWig files displaying the full length for uniquely mapping reads were generated using the bedGraphToBigWig (UCSC binary utilities). To investigate the co-occupancy of ADNP, BRG1 and CHD4, we consulted previously published ChIP-seq data sets for BRG1 (GSE87820) and CHD4 (GSE64825) (Dieuleveult et al., 2016). To investigate the co-occupancy of ADNP with H3K4me1 and H3K27ac, we consulted previously published ChIP-seq data sets for H3K4me1 (GSM2575694) and H3K27ac (GSM2575695) (Dieuleveult et al., 2016).

### RNA-seq Analysis

All sequencing reads were aligned to the 9 mm mouse genome assembly from the UCSC genome browser. Data were aligned using Bowtie2 with the default settings. Aligned and sorted reads were indexed using SAMtools (version 1.2). Reads were counted over exons using the R summarize Overlaps function and collapsed to yield one value per gene. The read counting is performed for exonic gene regions in a non-strand-specific manner while ignoring overlaps among different genes. Subsequently, the expression count values were normalized by Reads Per Kilobase per Million mapped reads (RPKM). The count table was used for differential expression calling with the EdgeR package. FDR < 0.05 and log_2_ fold change > 1 was deemed to be a differentially expressed gene. For comparative transcriptome analysis in the presence and absence of ADNP, BRG1 and CHD4, we consulted the published RNA-seq data sets for *Brg1* KO (GSE87821) and *Chd4* KO (GSE80280) (King and Klose, 2017).

### ATAC-seq Analysis

Paired-end reads were aligned using Bowtie2 using default parameters. Only uniquely mapping reads were kept for further analysis. These uniquely mapping reads were used to generate bigwig genome coverage files similar to ChIP–seq. Heat maps were generated using deeptools2. For the meta-profiles, the average fragment count per 10-bp bin was normalized to the mean fragment count in the first and last five bins, which ensures that the background signal is set to one for all experiments. Merged ATAC-seq datasets were used to extract signal corresponding to nucleosome occupancy information with NucleoATAC. For comparison analysis of ADNP, BRG1 and CHD4 ATAC-seq signals, we consulted previously published ATAC-seq data sets for *Brg1* KD (GSM1941485-6) and *Chd4* KD (GSM1941483-4) (Dieuleveult et al., 2016).

### Differential Binding and Gene Expression Analysis

Significant changes in ATAC-seq were identified using the DiffBind package, a FDR < 0.05 and log_2_ fold change > 1 was deemed to be a significant change. Gene ontology (GO) analysis for differentially regulated genes, and heat maps were generated from averaged replicates using the command line version of deepTools2. Peak centers were calculated based on the peak regions identified by MACS (see above).

### Quantification and Statistical Analysis

Data are presented as mean values ± SD unless otherwise stated. Data were analyzed using Student’s *t* test analysis. Error bars represent s.e.m. Differences in means were statistically significance when *p* < 0.05. Significant levels are: ^∗^*p* < 0.05; ^∗∗^*P* < 0.01; ^∗∗∗^*p* < 0.001.

### Data Availability

All RNA-seq, ATAC-seq and ChIP-seq data have been deposited into the database at https://bigd.big.ac.cn/. The accession numbers are CRA001624 and CRA002148. All other related data will be available upon reasonable request.

## Results

### *Adnp* Ablation Leads to Significant Up-Regulation of PrE Genes

**FIGURE 1:**
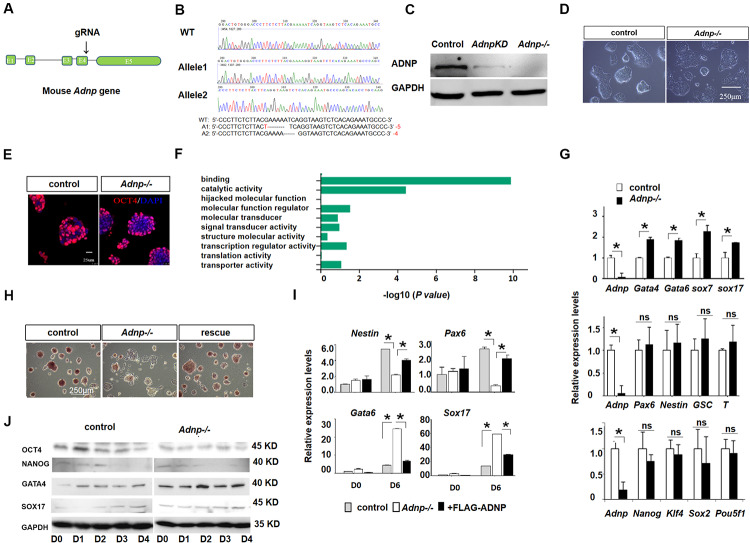
*Adnp* ablation leads to significant up-regulation of PrE genes. **(A)** Schematic representation of the mouse *Adnp* gene, depicting the location of the CRISPR/Cas9 targeting site. **(B)** Genotyping results of two mutant alleles. **(C)** WB assay of ADNP for *Adnp* knockdown and *Adnp-/-* ESCs. **(D)** Phenotypes of wild type and *Adnp-/-* ESCs. **(E)** Immunofluorescence assay of OCT4 for control and *Adnp-/-* ESCs. **(F)** GO analysis of DEGs in the absence of ADNP. **(G)** qRT-PCR assay of pluripotency, endodermal, neuroectodermal and mesodermal genes. **(H)** Alkaline phosphatase stain of wild type, *Adnp-/-* and FLAG-ADNP rescued ESCs. **(I)** Expression of key PrE and neural genes of day 0 and day 6 EBs derived from control and mutant ESCs. **(J)** WB showing the abnormal induction of SOX17 and GATA4 levels in the absence of ADNP. The RNA-seq, qRT-PCR and WB assays were based on three replicates. Differences in means were statistically significant when *p* < 0.05. Significant levels are: *p < 0.05.

To understand the role of ADNP, we generated *Adnp* mutant ESCs by using CRISPR/Cas9 technology. gRNAs were designed to target the 3′ end of exon 4 of the mouse *Adnp* gene (Figure 1A). We have successfully generated 4 *Adnp* mutant alleles. The mutant ESCs we used in this work has the combination of 4- and 5-bp deletions in exon 4 of *Adnp*, as revealed by DNA genotyping around the CRISPR targeting site (Figure 1B). ADNP protein was almost undetectable in *Adnp*-/- ESCs by Western blot using ADNP antibodies from different resources (Figure 1C and Supplementary Figure 1A), which strongly supported that the mutant alleles are functional nulls.

In the traditional self-renewal medium containing LIF-KSR plus FBS, the newly established *Adnp-/-* ESC colonies overall exhibited typical ESC-like morphology, and abundantly expressed the core pluripotency factor OCT4 (Figures 1D,E). To understand how ADNP deficiency affects ES cell phenotype, comparative transcriptome analysis for control and early passage *Adnp-/-* ESCs was performed by RNA-sequencing (RNA-seq). A total of 1,026 differentially expressed genes (DEGs) (log_2_ fold change > 1 and *p* < 0.05) were identified based on two RNA-seq experiments (each has two replicates). Of which, an average of 766 genes were up-regulated and 260 genes were down-regulated (Supplementary Figure 1B). GO (gene ontology) analysis revealed that the majority of deregulated genes were enriched for DNA binding and catalytic activity (Figure 1F). In the absence of ADNP, the expression of key mesoderm specifying genes such as *Gsc* and *T* (log_2_ fold change < 1; RPKM < 1 in ESCs), and neuroectoderm specifying genes such as *Fgf5*, *Nestin* and *Olig2* (log_2_ fold change < 1; RPKM < 2), as well as pluripotency genes such as *Pou5f1* and *Nanog* (log_2_ fold change < 1) was barely changed. Remarkably, genes implicated in extraembryonic primitive endoderm (PrE) development such as *Gata4*, *Gata6*, *Sox7*, *Krt18*, *Sparc*, *Cited1*, *Dab2*, and *Cubn* (log_2_ fold change > 1; RPKM > 5 in mutant ESCs) were significantly up-regulated. The qRT-PCR assay confirmed the RNA-seq results (Figure 1G). These data suggested that ADNP performs an important role in repressing PrE genes in ESCs. Although PrE genes were up-regulated, *Adnp-/-* ESCs can maintain self-renewal capacity for many generations before eventually adopting a flattened morphology and exhibiting reduced alkaline phosphatase activity (Figure 1H). Thus, our results indicated that acute ADNP depletion in ESCs does not result in sudden and complete loss of self-renewal, while prolonged ADNP depletion may cause ESC differentiation toward endodermal cell types, likely due to up-regulation of the key endoderm-specifying genes.

It has been shown that loss of ADNP disrupted the differentiation potential of ESCs (Ostapcuk et al., 2018). Similar results were obtained in our hand by performing embryoid body (EB) formation of mutant and control ESCs (Figure 1I). In day 6 EBs derived from control ESCs, neural genes *Nestin* and *Pax6* as well as PrE genes *Gata6* and *Sox17* were induced as expected. In day 6 EBs derived from *Adnp-/-* ESCs, however, the PrE genes were abnormally up-regulated, at the expense of neural genes. WB analysis confirmed that GATA6 and GATA4 levels were higher in *Adnp-/-* ESC-derived EBs than in control ESC-derived EBs (Figure 1J). When FLAG-ADNP was re-introduced into mutant ESCs (Supplementary Figure 1C), the defective gene expression and the alkaline phosphatase activity were largely rescued (Figures 1H,I). This data demonstrated that the observed phenotypes were specifically due to the loss of ADNP.

### ADNP Associates With Chromatin Remodelers BRG1 and CHD4

**FIGURE 2:**
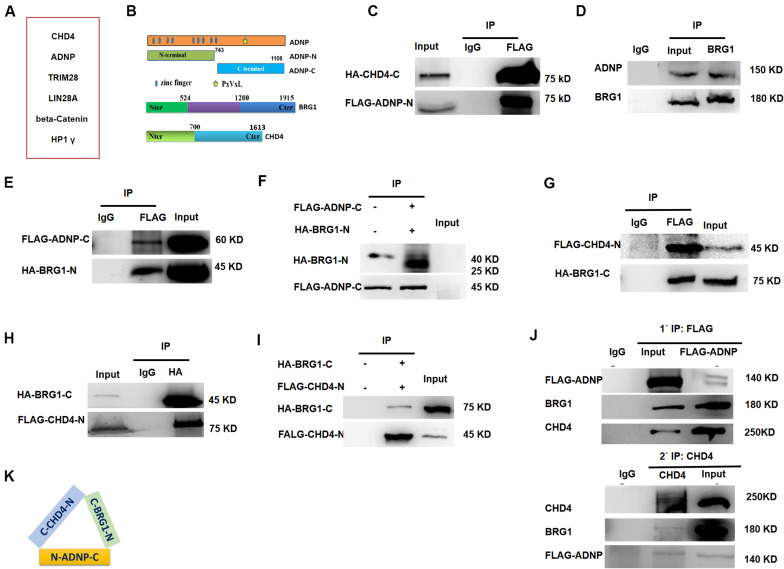
ADNP forms complexes with BRG1 and CHD4. **(A)** A table showing representative ADNP interacting proteins by IP followed mass-spectrometry assay. **(B)** Schematic representation of wild type ADNP, BRG1 and CHD4, and their truncated mutant forms. **(C)** FLAG-tagged ADNP-N pulled down HA-tagged CHD4-C in 293T. **(D)** Endogenous BRG1 pulled down ADNP in ESCs. **(E)** FLAG-tagged ADNP-C pulled down HA-BRG1-N in 293T. **(F)** FLAG-tagged ADNP-C pulled down HA-BRG1-N using a TNT system. **(G)** FLAG-CHD4-C pulled down HA-BRG1-N in 293T cells. **(H)** HA-BRG1-N pulled down FLAG-CHD4-C in 293T. **(I)** FLAG-tagged pulled down HA-BRG1-N using a TNT system. **(J)** Sequential IP showing that ADNP, CHD4 and BRG1 could form a triplex in ESCs. **(K)** A cartoon showing the interaction of all three factors, based on the mapping results. All WB and IPs were repeated at least two times. Shown are representative images.

To understand the role of ADNP in ESCs, we sought to identify its interacting proteins by performing immunoprecipitation (IP) in combination with mass spectrometry (Mass Spec) assay using commercial ADNP antibodies (Figure 2A). The commercial ADNP antibodies we used are specific, as demonstrated by the fact that a clean band around 150 kD which is the predicted size of ADNP was detected by WB analysis, and this band became barely detected in *Adnp-/-* ESCs (Figure 1C). A total of 180 ADNP-interacting candidate proteins were identified, which included the known ADNP interactors HP1γ and CHD4 (Ostapcuk et al., 2018), which further supported the specificity of the antibodies used. We confirmed that the N-terminal fragment of ADNP binds to CHD4 which was in line with the recent report by Ostapcuk and co-workers (data not shown). Further mapping experiments revealed that the N-terminal fragment of ADNP binds to the C-terminal but not N-terminal fragment of CHD4 (Figures 2B,C and Supplementary Figure 2A).

Activity Dependent Neuroprotective Protein was previously shown to interact with BRG1 and BAF250, core sub-units of BAF ATP-dependent chromatin remodeling complexes in HEK293 cells (Mandel and Gozes, 2007). BRG1 and BAF250 are conserved components of the ES cell-specific BAF complex called esBAF (Ho et al., 2008). Although no esBAF components were identified in our Mass Spec assay, *Adnp-/-* ESCs resemble BRG1 or BAF250a deficient ESCs not only in gene expression but also in morphological aspects (Gao et al., 2008; Kidder et al., 2009). We therefore performed co-IP experiments to examine whether ADNP interacts with BRG1 or BAF250a in ESCs. Our co-IP results showed that endogenous BRG1 but not BAF250a was able to pull down ADNP (Figure 2D). Further mapping experiments showed that the C-terminal fragment of ADNP interacts with the N-terminal but not the C-terminal of BRG1 (Figure 2E and Supplementary Figure 2B). To investigate whether ADNP physically associates with BRG1, we used a reticulate lysate system to synthesize the FLAG-tagged C-terminal fragment of ADNP and the HA-tagged N-terminal fragment of BRG1. When they were mixed together, anti-FLAG antibodies could readily pull down HA-BRG1-N (Figure 2F).

Although endogenous BRG1 and CHD4 interact with each other in mouse embryos, it is not known whether this was the case in ESCs (Shimono et al., 2003; Singh et al., 2016). By performing co-IP experiments, we found that endogenous BRG1 pulled down CHD4. Further mapping revealed that the C-terminal fragment of BRG1 strongly associates with the N-terminal fragment of CHD4 (Figures 2G,H). Using a reticulate lysate system, we further confirmed that they interact physically (Figure 2I).

Based on the above mapping results, we speculated that ADNP, CHD4, and BRG1 may form a triplex *in vivo*. To this end, we performed sequential immunoprecipitation experiments using a transgenic *Adnp-/-* ES cell line where a 3 × FLAG-tagged version of ADNP could be induced by the addition of the Tet-Express protein. We confirmed that in the presence of the Tet-Express protein, 3 × FLAG-ADNP levels in *Adnp-/-* ESCs were similar to endogenous ADNP in control ESCs (Supplementary Figure 2C). In the first round IP, FLAG antibodies easily pulled down endogenous BRG1 or CHD4. Next, FLAG antibody-bound protein complexes were eluted with excessive 3 × FLAG peptide, and were subjected to the second round of IP using CHD4 antibodies. As shown in Figure 2J, CHD4 antibodies could pull down both FLAG-ADNP and BRG1. Thus, our sequential IP data supported that ADNP, BRG1 and CHD4 could form a tripartite complex (ABC triplex) in ESCs (Figure 2K), although it is possible that this triplex is a part of large uncharacterized multiprotein complexes.

### ADNP, BRG1, and CHD4 Co-occupy Target Genes Genome-Wide

**FIGURE 3:**
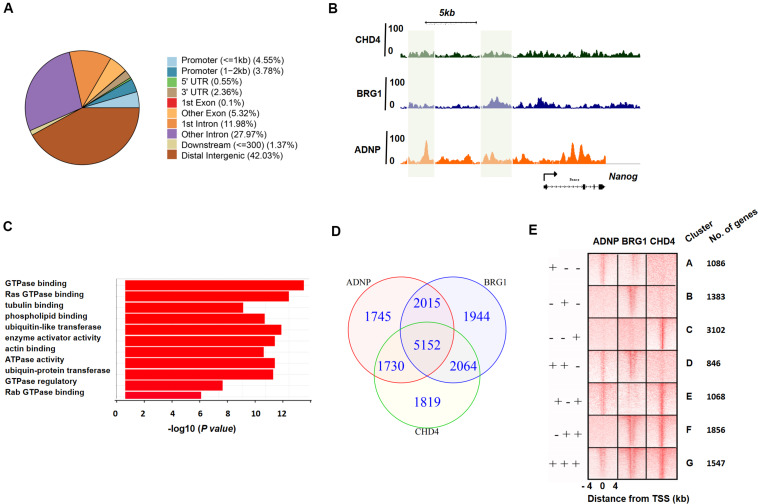
ADNP, BRG1 and CHD4 co-occupy target genes genome-wide. **(A)** Annotation of ADNP peaks genome-wide. **(B)** A snapshot of ChIP-seq genome-browser view of ADNP, BRG1 and CHD4 occupancy at the *Nanog* locus. The co-localized loci by all three factors were highlighted in green. **(C)** GO analysis of ADNP target genes. **(D)** A Venn diagram analysis of ChIP-seq peaks for ADNP, BRG1 and CHD4. **(E)** A heat map illustrating distribution of ADNP, BRG1 and CHD4 in ± 4 kb genomic regions around transcription start sites (TSS), with gene cluster assignment and the number of each clusters.

To better understand the role of ADNP in the maintenance of ESCs, we sought to determine its direct targets and genome-wide binding profile by chromatin immunoprecipitation coupled with high-throughput sequencing (ChIP-seq) analysis. A total of 10,642 sites were bound by ADNP compared to the input, and 838 target genes were identified. Of which, 1,632 peaks were found in promoter proximal regions, 5,951 peaks were found in gene bodies, and the majority of the remainder were localized to intergenic regions (Figures 3A,B and Supplementary Figures 3A,B). Thus, most of ADNP peaks were localized to intergenic or promoter-distal regions, which was similar to that from the recent published FLAG-ADNP ChIP-seq results (Ostapcuk et al., 2018). Gene ontology analysis revealed that ADNP targets are enriched for genes involved in metabolic processes and cell signaling such as GTPase binding, G-protein signaling and cell adhesion (Figure 3C). As PrE genes were significantly deregulated in the absence of ADNP, we examined ADNP ChIP-seq peaks at these genes. Surprisingly, no significantly enriched ChIP-seq peaks were found at key PrE genes except *Gata4* (Supplementary Figure 3B). By contrast, pluripotency genes such as *Nanog* and *Pou5f1* were extensively bound by ADNP (Figure 3A). By ChIP-PCR, we confirmed that *Gata6*, *Sox7* and *Sox17* were barely bound by ADNP at gene promoter regions (Supplementary Figure 3C).

The association of ADNP with BRG1 and/or CHD4 prompted us to determine whether ADNP binding sites were co-occupied by the two chromatin remodeler factors. Unfortunately, the ChIP experiments using commercial BRG1 or CHD4 antibodies were not successful. We therefore consulted the published CHD4 or BRG1 ChIP-seq data, and revealed that 10,765 and 11,175 sites were significantly enriched for CHD4 and BRG1, respectively (Dieuleveult et al., 2016; King and Klose, 2017). CHD4 or BRG1 ChIP-seq peaks were localized to proximal promoter, gene body and intergenic regions, analogous to that of ADNP. Bioinformatics analysis were performed to examine the overlap among ADNP, BRG1 and CHD4 ChIP-seq peaks. When we compared ADNP and BRG1 sites, 67% (7,167/10,642) of ADNP peaks overlapped with 64% (7,167/11,175) of BRG1 peaks; when comparing ADNP with CHD4 sites, 65% (6,882/10,642) of ADNP peaks overlapped with 64% of CHD4 peaks (6,882/10,765). When comparing the binding of all three proteins, 31% (5,152/16,469) were co-bound by ADNP, BRG1 and CHD4 (Figures 3B,D and Supplementary Figure 3A).

We plotted ADNP, CHD4, and BRG1 ChIP-seq reads in a ± 4 kb region surrounding TSS and divided ADNP- or CHD4- or BRG1-bound genes into 7 categories (cluster A: ADNP+BRG1- CHD4-, cluster B: ADNP-BRG1+ CHD4-, cluster C: ADNP-BRG1-CHD4+, Cluster D: ADNP+BRG1+ CHD4-, cluster E: ADNP+BRG1-CHD4+, cluster F: ADNP-BRG1+CHD4+, and cluster G: ADNP+ CHD4+ BRG1+) (Figure 3E). We examined the effects of loss of ADNP on the expression of each cluster of genes. Interestingly, compared to all genes, loss of ADNP had a minimal effects on gene expression of all clusters except for cluster D (Supplementary Figure 3D). Loss of ADNP led to a significant down-regulation of cluster D genes (*p* < 0.05). GO analysis of cluster D revealed the enrichment of terms such as regulation of transcription, positive regulation of neural differentiation, cell cycle and metabolic process (data not shown). This was in line with that loss of ADNP leads to compromised ESC pluripotency, particularly differentiation toward the neuronal lineage (Ostapcuk et al., 2018). Why the cluster D genes were most sensitive to loss of ADNP remains unclear.

### ADNP Depletion Leads to Local Chromatin Accessibility and Nucleosome Configuration Change, and PrE Genes Appear Most Sensitive to Loss of *Adnp*

**FIGURE 4:**
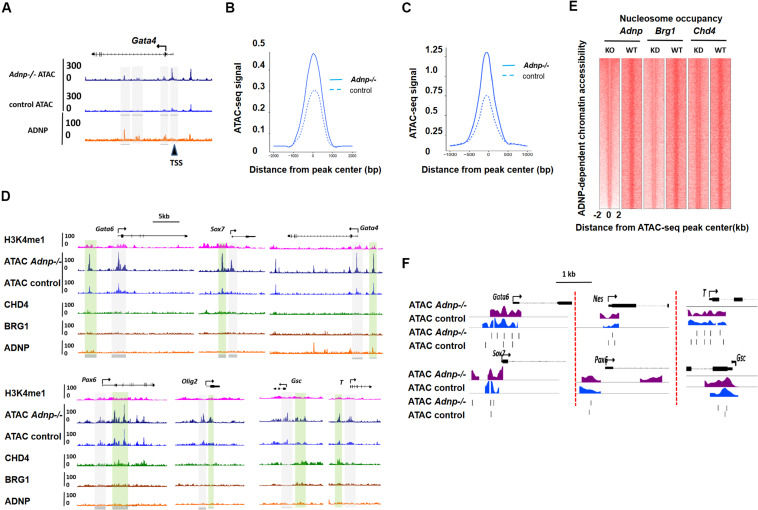
ADNP depletion leads to chromatin accessibility and nucleosome configuration change. **(A)** A genomic snapshot of ATAC-seq and ADNP ChIP-seq signal at the *Gata4* locus in control and *Adnp-/-* ESCs. Gray color highlighting the ADNP-dependent ATAC-seq peak loci. Black triangle indicating the significant increase of ATAC-seq signal at TSS region where no ADNP ChIP-seq signal was detected. **(B)** A metaplot of average ADNP-dependent ATAC-seq signal in control and *Adnp-/-* ESCs. **(C)** A metaplot of average ATAC-seq signal at TSS regions in control and *Adnp-/-* ESCs. Similar results were obtained at enhancer regions (not shown). **(D)** A genomic snapshot of ADNP, BRG1, CHD4 and H3k4me1 ChIP-seq and ATAC-seq at lineage specifying genes in control and *Adnp-/-* ESCs. H3K4me1 was used for showing the poised enhancer regions. Gray color highlighting TSS-proximal regions, and green color highlighting enhancer regions. Except for *Gata4*, no key lineage specifying genes were bound by ADNP. Chromatin accessibility was increased at TSS-proximal and enhancer regions. **(E)** Nucleosome occupancy around ATAC-seq peak center in control, *Adnp* knockout, *Brg1* knockdown and *Chd4* knockdown ESCs. **(F)** A snapshot of nucleosome configuration at TSS region of representative lineage-specifying genes in control and *Adnp-/-* ESCs. ATAC-seq experiments were repeated two times for control and *Adnp-/-* ESCs. ATAC-Seq data for BRG1 and CHD4 were downloaded as described in the text. All analysis was based on the two replicates.

Activity Dependent Neuroprotective Protein interacting chromatin remodelers CHD4 and BRG1 have well-known functions for regulating chromatin accessibility and nucleosome configuration in ESCs (Tolstorukov et al., 2013; Lei et al., 2015). To understand how loss of ADNP affected gene expression, we performed transposase-accessible chromatin with massively parallel sequencing (ATAC-seq) for control and *Adnp-/-* ESCs. In control ESCs, the majority of ADNP-bound loci were largely devoid of ATAC-seq signals, suggesting that ADNP was bound to inaccessible chromatin. In the absence of ADNP, these sites became accessible as they showed significant ATAC-seq signals (Supplementary Figures 4A,B). The *Gata4* gene is shown here for individual representation (Figure 4A). This observation was in line with the recent report that ADNP may render local chromatin inaccessible by directly binding to these loci (Ostapcuk et al., 2018). Remarkably, we found that loss of ADNP also caused a widespread increase of ATAC-seq signals at genome loci where weak or no ADNP ChIP-seq signals were observed, primarily at gene enhancer and proximal-TSS regions (Figures 4A–D). This observation suggested that ADNP functions to restrict chromatin accessibility at gene regulatory regions, through a mechanism independent of its DNA binding activities. Alternatively, the chromatin accessibility at gene regulatory regions is very sensitive to loss of ADNP.

Specifically, we compared chromatin accessibility for endoderm, mesoderm and neuroectoderm specifying genes in the presence and absence of ADNP. In the absence of ADNP, a substantial increase of chromatin accessibility at both proximal-TSS and poised enhancer regions was observed for key endoderm specifying genes such as *Gata6* and *Sox7* (Figure 4D). Chromatin accessibility was also changed for key mesoderm and neuroectoderm specifying genes.

Next, we asked whether ADNP regulates nucleosome configuration in ESCs. Globally, nucleosome occupancy was significantly reduced in the absence of ADNP (Figure 4E). When examining the key lineage-specifying genes, we found that nucleosome positioning, phasing and occupancy were all significantly altered in the absence of ADNP (Figure 4F). It appeared that loss of ADNP had greater effects on nucleosome configuration for the PrE genes than mesoderm and neuroectoderm specifying genes: ADNP depletion led to a significant nucleosome occupancy increase around the TSS of PrE genes. Of note, the nucleosome configuration of ADNP-bound pluripotency genes was barely altered in the absence of ADNP (Supplementary Figure 4C).

### ADNP-Regulated Chromatin Mechanism Is Linked With BRG1 and CHD4

Chromatin remodelers are well-known for their role in the regulation of chromatin structure (Musselman et al., 2012; Clapier et al., 2017; Wang et al., 2017). Based on the observation that ADNP, CHD4, and BRG1 could form complexes and co-occupy target genes, we reasoned that an ADNP-regulated chromatin mechanism might be linked with BRG1 and CHD4.

**FIGURE 5:**
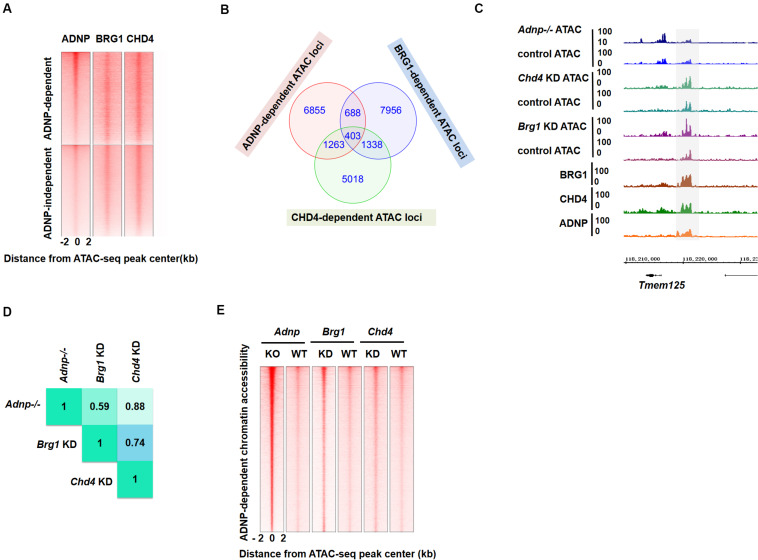
BRG1 and CHD4 are closely-linked with ADNP-regulated chromatin accessibility. **(A)** Heatmap showing ADNP, BRG1 and CHD4 ChIP-seq enrichment around ± 2 kb ADNP-dependent and ADNP-independent ATAC-seq peak centers. **(B)** A Venn diagram analysis of BRG1-, CHD4- and ADNP-dependent ATAC hypersensitive peaks. **(C)** A snapshot showing overlapping BRG1-, CHD4- and ADNP-dependent ATAC-seq peaks at the *Tmem125* locus. **(D)** Correlation analysis of loci with overlapping ATAC-seq signals revealed a high degree of co-localization of BRG1-, CHD4- and ADNP-dependent ATAC hypersensitive peaks. **(E)** A heat map of ATAC-seq signal around the ATAC peak center, in the presence or absence of each factor. Sites are ranked by the increase of ATAC-seq signal following loss of ADNP activity.

To explore this, we plotted ADNP ATAC-seq with ADNP, BRG1, and CHD4 ChIP-seq data sets, and asked whether ADNP-dependent ATAC hypersensitive peaks overlapped with BRG1 or CHD4 ChIP-seq peaks. Indeed, we found that ADNP, CHD4 or BRG1 ChIP-seq peaks partially overlapped with ADNP-dependent ATAC hypersensitive loci (Figure 5A). Of note, ADNP ChIP-seq signals were stronger at ADNP-dependent than ADNP-independent ATAC-seq peak loci. This was not the case for BRG1 and CHD4 ChIP-seq signals. This observation suggested that there is an inherent functional link among ADNP, BRG1 and CHD4 in shaping chromatin accessibility, and that ADNP may use both CHD4 and BRG1 to regulate chromatin accessibility by binding to the local genomic loci.

To further investigate the above hypothesis, we consulted the previously published BRG1 or CHD4 ATAC-seq data sets (King and Klose, 2017). We plotted BRG1, CHD4, and ADNP ATAC-seq data reads, and examined the co-localization of ATAC hypersensitive peaks in the absence of each factor. We found that about 10% of BRG1- or 17% of CHD4-dependent ATAC hypersensitive peaks overlapped with ADNP-dependent ATAC hypersensitive peaks (Figures 5B,C). Correlation analysis of loci with overlapping ATAC-seq signals revealed a high degree of co-localization of BRG1-, CHD4- and ADNP-dependent ATAC hypersensitive peaks (Figure 5D). Interestingly, ADNP depletion exhibited a much stronger effect on ATAC-seq signal than either BRG1 or CHD4 depletion at these loci (Figure 5E). These observations suggested that a co-dependency of BRG1 and CHD4 mediated by ADNP may be utilized to regulate chromatin architecture.

To determine the potential contribution of the two distinct chromatin remodelers, we compared the change of ATAC-seq signal in the absence of ADNP, BRG1 and CHD4, for developmental genes, especially the PrE-related genes. We observed that chromatin accessibility at a substantial fraction of genome loci was affected by all three factors (Supplementary Figure 5A). For PrE genes such as *Foxa2* and *Sparc*, either ADNP- or BRG1- or CHD4-depletion led to increased ATAC-seq signal, suggesting that BRG1 and CHD4 activities are synergistically required to maintain a closed chromatin architecture. For PrE genes such as *Sox7* and *Gata4*, chromatin accessibility was predominantly affected by ADNP and CHD4 as ATAC-seq signal was not altered by BRG1 depletion. For neuroectodermal genes such as *Fgf5* and *Nestin*, loss of BRG1 led to a reduction of ATAC-seq signal while CHD4 or ADNP depletion led to an increase of ATAC-seq signal, suggesting that BRG1 and CHD4 act antagonistically.

### Loss of ADNP Caused Significant Change of Bivalent Histone Modifications for Developmental Genes

**FIGURE 6:**
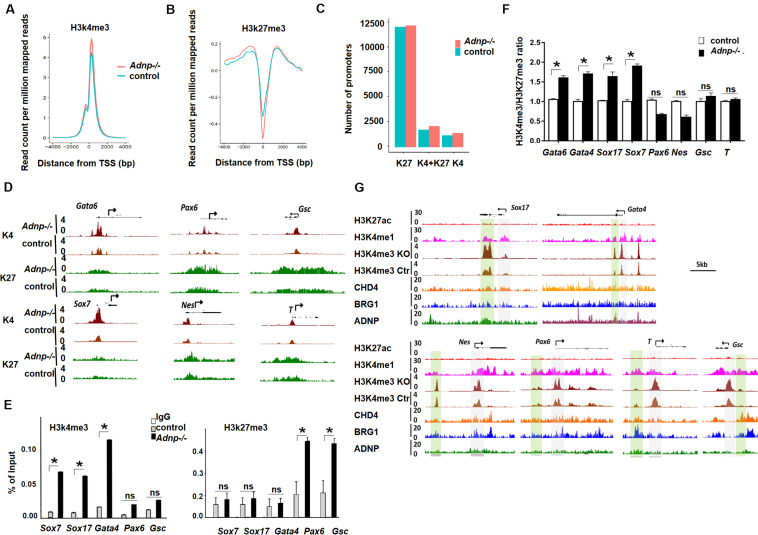
Loss of ADNP caused histone modification change genome-wide. **(A)** A metaplot analysis of H3K4me3 occupancy at the TSS regions of all genes in control and *Adnp-/-* ESCs. **(B)** A metaplot analysis of H3K27me3 occupancy at TSS region of all genes in control and *Adnp-/-* ESCs. **(C)** Change of gene numbers of three gene clusters in the absence of ADNP. **(D)** A genomic snapshot of H3K4me3 and H3K27me3 ChIP-seq peaks at the indicating loci. **(E)** ChIP-PCR assay of H3K4me3 and H3K27me3 at the indicated gene promoters. **(F)** A quantitation of H3K4me3/H3K27me3 ratio at indicated gene promoters based on two replicates of H3K4me3 and H3K27me3 ChIP-seq data. **(G)** Genome browser view at the lineage specifying genes in the control and *Adnp-/-* ESCs. H3K4me1 ChIP signal was used for showing the poised enhancers. H3K27ac ChIP signal was used for the active enhancers. Gray color highlighting TSS-proximal regions, and green color highlighting enhancer regions. Note, H3K4me3 levels were substantially elevated at enhancer region of PrE but not for mesodermal and ectodermal genes. H3K4me3 and H3K27me3 ChIP-seq were repeated two times. Differences in means were statistically significant when *p* < 0.05. Significant levels are: *p < 0.05.

It has been known that ADNP-interacting chromatin remodelers BRG1 and CHD4 contribute to the establishment of bivalent histone modifications (Tolstorukov et al., 2013; Lei et al., 2015). We asked whether loss of ADNP led to the alteration of bivalent histone modifications for developmental genes in ESCs. To investigate this, we performed ChIP-seq analysis for H3K4me3 and H3K27me3 of control and *Adnp-/-* ESCs. Bioinformatics analysis of the ChIP-seq data showed that the levels of both H3K4me3 and H3K27me3 were changed by loss of ADNP (Figures 6A,B). We grouped gene promoters into three categories: H3K4me3 only, H3K27me3 only, and both H3K4me3 and H3K27me3, and asked how the histone marks changed in each category in the absence of ADNP. Bioinformatics analysis revealed that loss of ADNP caused a significant increase of both H3K4me3 (around the TSS) and H3K27me3 (0.5–4 kb upstream the TSS), resulting in a slightly increased number of all three cluster of promoters (Figure 6C and Supplementary Figures 6A,B).

To understand why loss of ADNP was associated with an up-regulation of PrE genes, we examined bivalent histone modifications for lineage-specifying genes in control and *Adnp-/-* ESCs. It seems that loss of ADNP had different effects on bivalent histone modifications depending on the lineage-specifying genes. For instance, at mesodermal genes such as *T* and *Gsc*, and neuroectodermal genes such as *Olig2*, *Pax6* and *Nestin*, a slight increase of both H3K4me3 and H3K27me3 levels was observed in *Adnp-/-* ESCs compared with control ESCs. At PrE specifying genes such as *Gata6*, *Gata4*, *Sox17*, *Sox7* and *Foxa2*, a substantial increase of H3K4me3 levels was observed, while H3K27me3 levels were slightly increased (Figure 6D). We confirmed this by ChIP-PCR (Figure 6E). It is known that the levels of H3K4me3 correlate with gene activation, and the levels of H3K27me3 correlate with gene repression. And there is a positive correlation between transcript levels and H3K4me3/H3K27me3 ratio for bivalent genes in pluripotent stem cells (De Gobbi et al., 2011; Singh et al., 2015). We therefore compared the H3K4me3/H3K27me3 ratio for key lineage-specifying genes in *Adnp-/-* and control ESCs. A significant increase of H3K4me3/H3K27me3 ratio was observed at promoters of PrE genes such as *Gata6* and *Sox7* whose expression were prominently up-regulated in the absence of ADNP. For genes such as *T* and *Gsc* whose expression was barely changed in the absence of ADNP, the H3K4me3/H3K27me3 ratio in mutant ESCs was comparable to that of control ESCs (Figure 6F). Thus, loss of *Adnp* caused a significant increase of the H3K4me3/H3K27me3 ratio for key PrE specifying genes but not for mesodermal or neuroectodermal genes.

It is well-known that the accurate execution of gene expression programs requires 2 types of regulatory DNA elements in higher eukaryotes: promoters and enhancers. We previously showed that promoter-enhancer interactions play important roles in 3D genome organization and the control of gene expression in ESCs (Phillips-Cremins et al., 2013; Singh et al., 2015). To further understand the role of ADNP in the regulation of gene expression, we investigated how loss of ADNP affected enhancer activities of key lineage specifying genes. We found that there was a substantial increase of H3K4me3 at poised enhancer regions of PrE but not mesodermal or neuroectodermal genes in the absence of ADNP (Figure 6G). It has been suggested that enhancer over-activation correlates with increased H3K4me3 and decreased H3K4me1 levels (Shen et al., 2016). Thus, our data suggested that ADNP is required to maintain poised enhancers for PrE developmental genes, and loss of ADNP leads to enhancer over-activation.

MLL2 is the core component of the MLL complex that deposits H3K4me3, and EZH2 is the core component of the PRC2 complex that deposits the H3K27me3 mark at bivalent promoters (Ku et al., 2008). We investigated whether ADNP depletion affected MLL2 or EZH2 binding at gene promoters by performing MLL2 or EZH2 ChIP-PCR experiments. We found that MLL2 levels at *Sox7, Gata4* and *Gata6* promoters were significantly elevated in *Adnp-/-* ESCs compared with control ESCs, while EZH2 enrichment was significantly enhanced at *Nestin* and *Pax6* promoters (Supplementary Figures 6C,D). Consistently, RNA polymerase II (Pol II) was significantly elevated at *Sox17* and *Gata6* but not at *Gsc* and *Pax6* genes (Supplementary Figure 6E).

## Discussion

In this work, we show that ADNP functions as an important chromatin regulator or genome organizer by association with two distinct chromatin regulators, BRG1 and CHD4. ADNP, BRG1 and CHD4 are extensively co-localized genome-wide and they cooperatively control chromatin accessibility and nucleosome configuration. Loss of ADNP expression leads to significant change of nucleosome landscape, bivalent histone modifications and enhancer activities of PrE genes, resulting in de-repression of these genes and priming ESCs differentiation into endodermal cell types.

While this work was ongoing, Ostapcuk et al. (2018) reported a similar study showing that ADNP controls lineage-specifying genes by forming complex with HP1 and CHD4. In their work, it appeared that loss of ADNP had immediate effects on ES cell phenotype. This was demonstrated by a grossly abnormal ESC morphology, reduced alkaline phosphatase activities, deregulation of lineage-specifying genes and reduced expression of pluripotency genes of *Adnp-/-* ESCs. However, in our hands, acute ADNP depletion in ESCs does not result in sudden and complete loss of self-renewal, and our *Adnp-/-* ESCs exhibit a milder phenotype compared to the counterpart ESCs described by Ostapcuk et al. First, our newly established *Adnp-/-* ESCs exhibited an ESC-like morphology and strong alkaline phosphatase activities. They could be passaged for many generations in the LIF/KSR medium. Only prolonged depletion of ADNP resulted in loss of ESC phenotype. Second, the RNA-sequencing analysis showed that the expression of pluripotency-related genes was barely changed in the newly established *Adnp-/-* ESCs. Third, the lineage-specifying genes were deregulated to a much lower extent when compared to that by Ostapcuk. For instance, the expression of *Igfbp4* and *Gsc* was not changed in the absence of ADNP in our study. The up-regulation of PrE genes was within 2–3-fold range, while this was over 5 times more in mutant cells by Ostapcuk. We think that the discrepancy could be due to the nature of the *Adnp* mutant alleles that were generated by Ostapcuk et al. and our group. In Ostapcuk’s work, a very large fragment of the *Adnp* gene (including exons 3 and 4, most of exon 5 as well as introns 3 and 4) was deleted. By looking up the UCSC genome browser, it is likely that there are putative enhancers in the deleted region of the *Adnp* gene from Ostapcuk et al. (2018) in ESCs, but this requires validation. In our work, only 4 or 5 bp deletion in exon 4 of *Adnp* gene was introduced, which should only disrupt *Adnp* gene function. Importantly, our rescue experiments showed that FLAG-tagged ADNP could largely restore the phenotypes of *Adnp-/-* ESCs which was not reported by Ostapcuk et al. (2018). As our CRISPR-Cas9 mediated base pair deletions resemble the human *ADNP* mutation (ID number 64, c.190dupA) in patients with HVDAS syndrome (Van Dijck et al., 2019), this work may help to explain the pleiotropic phenotypes (other than neurodevelopmental defects) observed in patients.

In this work, we reported that ADNP could form a ABC triplex with BRG1 and CHD4 in ESCs. It has been shown that ADNP forms a stable ChAHP triplex with CHD4 and HP1 (Ostapcuk et al., 2018). In addition, ADNP was shown to associate with components of the SWI/SNF complex in HEK293 cells (Mandel and Gozes, 2007). Thus, it appears that ADNP could form different complexes with certain factors depending on its cellular functions. We propose that the ChAHP and ABC triplexes in ESCs are not exclusive, and that ADNP may control local genome structure or chromatin accessibility by recruiting different chromatin remodelers or regulators.

An interesting observation is that loss of ADNP leads to significant up-regulation of a panel of PrE genes, indicating that ADNP is required to robustly repress PrE genes in an undifferentiated ESCs. It was known that the balance between SOX17/GATA6 and NANOG/OCT4 maintains ESC in undifferentiated state (Niakan et al., 2010; Wamaitha et al., 2015). Although ADNP binds to pluripotency genes such as *Nanog* and *Pou5f1*, its loss had little effect on the expression of these genes. Thus, ADNP cannot regulate the expression of PrE genes through repressing *Nanog*. Signaling pathways, such as FGF/Erk signaling, play key role in the expression of PrE genes (Chappell et al., 2013). However, the expression of key components of the signaling pathways was barely altered in the absence of ADNP. Based on these observations, we propose that ADNP contributes to gene expression primarily by regulating local chromatin structure. Several lines of evidence supports the notion. First, ADNP is known to maintain proper local chromatin architecture in ESCs (Kaaij et al., 2019). Loss of ADNP may directly or indirectly alter promoter-enhancer interaction frequencies and affect gene expression. Second, ADNP is important for the proper bivalent histone modifications at developmental gene promoters. An increased ratio of H3K4me3/H3K27me3 at key PrE gene promoters was observed in the absence of ADNP. Third, loss of ADNP leads to prominent enhancer over-activation of key PrE genes by increasing H3K4me3. Fourth, ADNP regulates nucleosome configuration genome-wide. In the absence of ADNP, nucleosome positioning, phasing and occupancy were all changed at a greater extent in PrE than mesodermal and neuroectodermal genes. A recent study showed that the proper nucleosome landscape plays an important role in the control of gene expression (King et al., 2019). Taken together, we propose that loss of ADNP leads to both enhancer over-activation and increased ratio of H3K4me3/H3K27me3 at gene promoters of PrE genes which may explain why *Adnp-/-* ESCs exhibited significant up-regulation of PrE genes.

Another intriguing observation is that although the majority of ADNP bound sites are associated with protein-coding genes, most ADNP ChIP-seq signals are not found at promoter-proximal regions (Kaaij et al., 2019). In addition, many genes bound by ADNP are not deregulated in the absence of ADNP. It appears that gene expression changes with the loss of ADNP are not predicted by ADNP binding. This further implies that ADNP controls gene expression by controlling local chromatin architecture, which is likely mediated by BRG1, CHD4 and CTCF (Lei et al., 2015; O’Shaughnessy-Kirwan et al., 2015; Kaaij et al., 2019). Thus, we propose that ADNP may control gene expression by binding to gene regulatory regions (as a transcription factor) and by association with BRG1, CHD4 and CTCF (as a genome organizer).

A previous study has shown that BRG1 and CHD4 co-occupy distal sites corresponding to increased ESC master TF binding, and that co-dependency of BRG1 and CHD7 exists to support pluripotency network in ESCs (Yang et al., 2017). This study suggested that concerted activities of multiple chromatin remodelers are utilized to support ES cell pluripotency. To our knowledge, whether and how distinct chromatin remodelers work cooperatively to modulate chromatin architecture to regulate lineage-specifying genes is not clear. In this work, we showed that a co-dependency of SWI/SNF-BRG1 and CHD4 may underlie for robust chromatin regulation for developmental genes. Thus, our work extends previous studies by showing that chromatin remodelers are cooperatively used not only for supporting core pluripotency genes, but also for silencing developmental genes while keeping them poised for activation.

## Data Availability Statement

The datasets generated for this study can be found in the CRA002148.

## Author Contributions

XS: collection of data and molecular biology experiments. WY: collection of data and bioinformatics analysis. LL: bioinformatics analysis and data interpretation. YS: conception and design, collection of data, manuscript writing, financial support, and final approval of manuscript. All authors contributed to the article and approved the submitted version.

## Conflict of Interest

The authors declare that the research was conducted in the absence of any commercial or financial relationships that could be construed as a potential conflict of interest.

## Supplementary Material

FIGURE S1 **(A)** shRNA knockdown of *Adnp* in ESCs. **(B)** Volcano plot showing the number of up- and down-regulated DEGs. **(C)** WB showing the FLAG-ADNP levels in *Adnp-/-* ESCs. All data were based on two experimental repeats.

FIGURE S2 **(A)** Synthesized FLAG-tagged ADNP-N failed to pull down MYC-tagged CHD4-C. **(B)** HA-BRG1-N pulled down full-length ADNP and FLAG-ADNP-C but not FLAG-ADNP-N in 293T cells. **(C)** WB showing that in the presence of Tet-Express protein, 3 × FLAG-tagged ADNP could be induced in *Adnp-/-* ESCs.

FIGURE S3 **(A)** A snapshot of ChIP-seq genome-browser view of ADNP, BRG1 and CHD4 occupancy around the *Vamp5* and *Nodal* loci, showing the co-localization of all three factors in green. **(B)** A snapshot of ChIP-seq genome-browser view of ADNP occupancy around the *Gata4/Gata6/Sox17* loci. **(C)** Enrichment of ADNP at the indicated gene promoters by ChIP-PCR assay using ADNP antibodies (IgG as negative control) based on two repeats. ns: no significance. **(D)** A piano plot showing the expression change of the indicated gene clusters in the absence of ADNP, based on two replicates of RNA-seq data.

FIGURE S4 **(A)** ChIP-seq genome browser view of ADNP ChIP-seq and ATAC-seq signals at part of chromosome 5 in control and *Adnp-/-* ESCs. Note the widespread increased ATAC signals in the absence of ADNP. **(B)** A example of ChIP-seq genome browser view of ADNP ChIP-seq and ATAC-seq signal at the *Dnahc8* locus. Gray: ATAC hypersensitive signal peaks were co-localized with ADNP ChIP-seq peaks. **(C)** Nucleosome configuration at promoter of the indicated pluripotency genes was not significantly altered in the absence of ADNP.

FIGURE S5 **(A)** A snapshot of ATAC-seq signal change at the indicating loci in the absence of each factor. ATAC-seq experiments were repeated two times for control and *Adnp-/-* ESCs. ATAC-Seq data for BRG1 and CHD4 were downloaded as described in the text.

FIGURE S6 **(A)** A metaplot analysis of H3K4me3 occupancy at TSS region of H3K4me3 only, H3K27me3 only and bivalent genes in control and *Adnp-/-* ESCs. **(B)** A metaplot analysis of H3K27me3 occupancy at TSS region of H3K4me3 only, H3K27me3 only and bivalent genes in control and *Adnp-/-* ESCs. The results were based on two replicates of H3K4me3 and H3K27me3 CHIP-seq experiments. **(C)** MLL2 enrichment at the indicated genes by ChIP-PCR assay. **(D)** EZH2 enrichment at the indicated genes by ChIP-PCR assay. **(E)** Pol II enrichment at indicated genes by ChIP-PCR analysis. All data were based on three repeat experiments. Differences in means were statistically significant when *p* < 0.05. Significant levels are: ^∗^*p* < 0.05; ^∗∗^*P* < 0.01; ^∗∗∗^*p* < 0.001.
