# Supplementary figures and images for: ADNP Controls Gene Expression Through Local Chromatin Architecture by Association With BRG1 and CHD4
Source: Front Cell Dev Biol. 2020 Jul 1;8:553. doi: 10.3389/fcell.2020.00553 (PMC7341970; doi:10.3389/fcell.2020.00553)

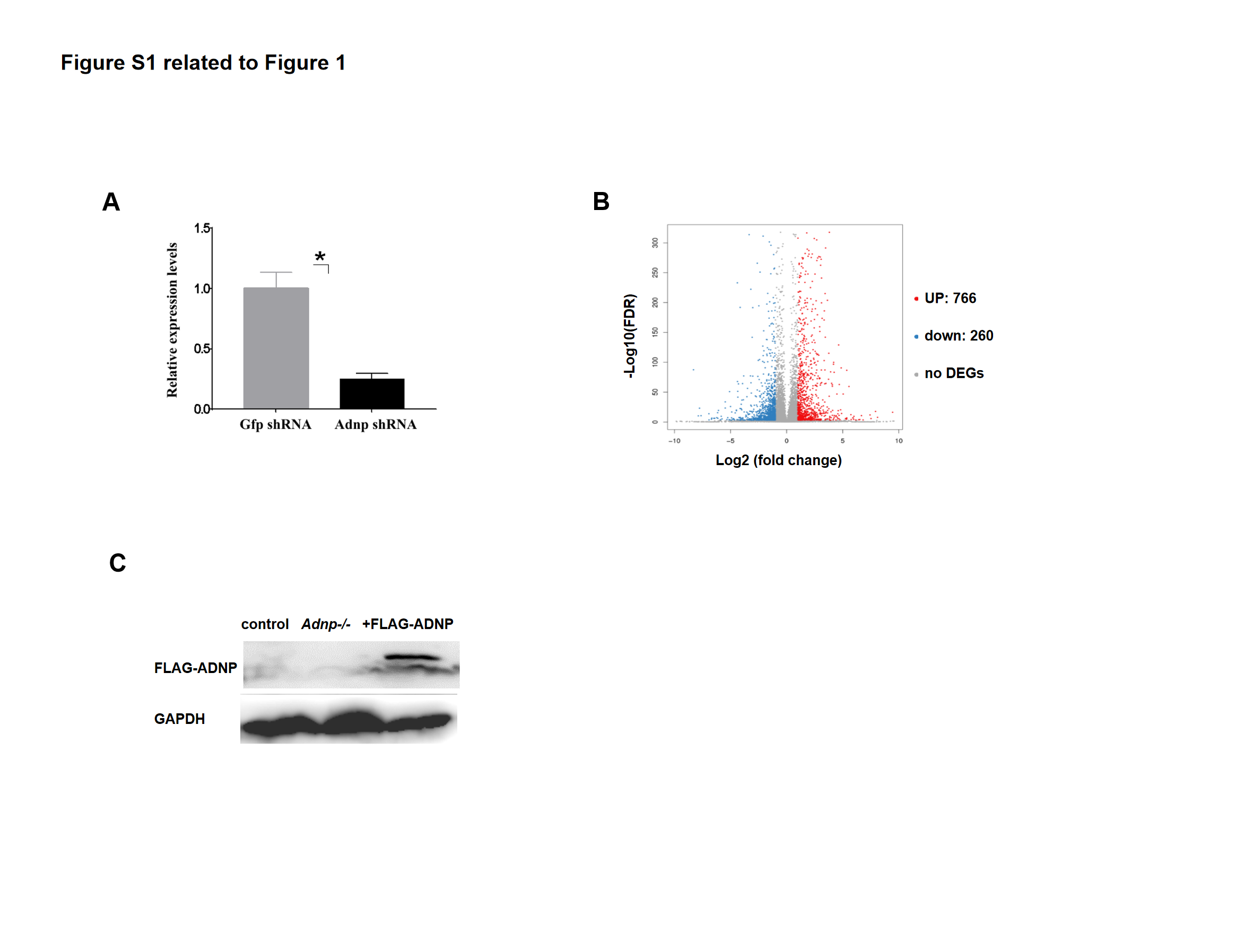

Supplement: FIGURE S1 — (A) shRNA knockdown of Adnp in ESCs. (B) Volcano plot showing the number of up- and down-regulated DEGs. (C) WB showing the FLAG-ADNP levels in Adnp-/- ESCs. All data were based on two experimental repeats. [file Image_1.TIF]

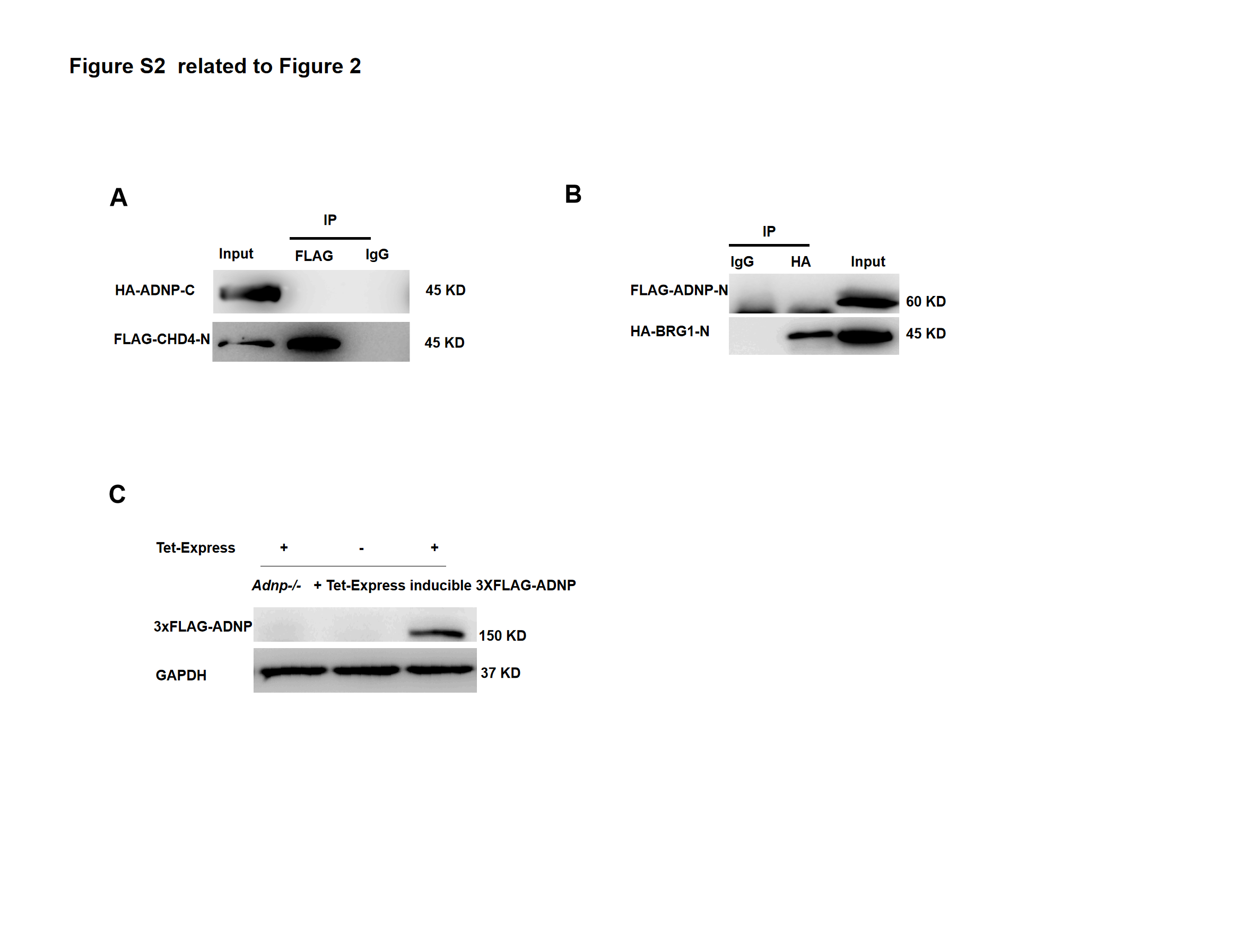

Supplement: FIGURE S2 — (A) Synthesized FLAG-tagged ADNP-N failed to pull down MYC-tagged CHD4-C. (B) HA-BRG1-N pulled down full-length ADNP and FLAG-ADNP-C but not FLAG-ADNP-N in 293T cells. (C) WB showing that in the presence of Tet-Express protein, 3 × FLAG-tagged ADNP could be induced in Adnp-/- ESCs. [file Image_2.TIF]

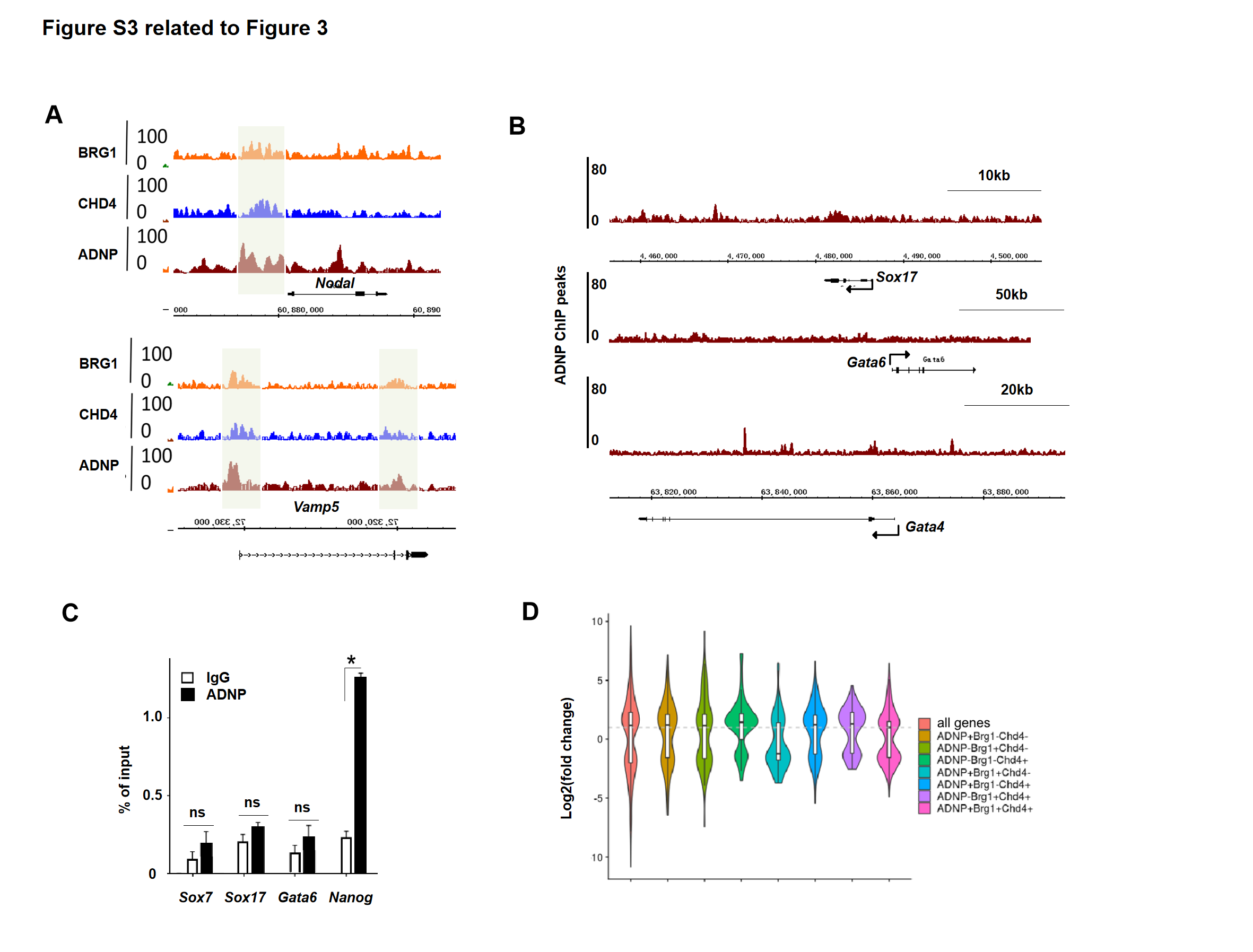

Supplement: FIGURE S3 — (A) A snapshot of ChIP-seq genome-browser view of ADNP, BRG1 and CHD4 occupancy around the Vamp5 and Nodal loci, showing the co-localization of all three factors in green. (B) A snapshot of ChIP-seq genome-browser view of ADNP occupancy around the Gata4/Gata6/Sox17 loci. (C) Enrichment of ADNP at the indicated gene promoters by ChIP-PCR assay using ADNP antibodies (IgG as negative control) based on two repeats. ns: no significance. (D) A piano plot showing the expression change of the indicated gene clusters in the absence of ADNP, based on two replicates of RNA-seq data. [file Image_3.TIF]

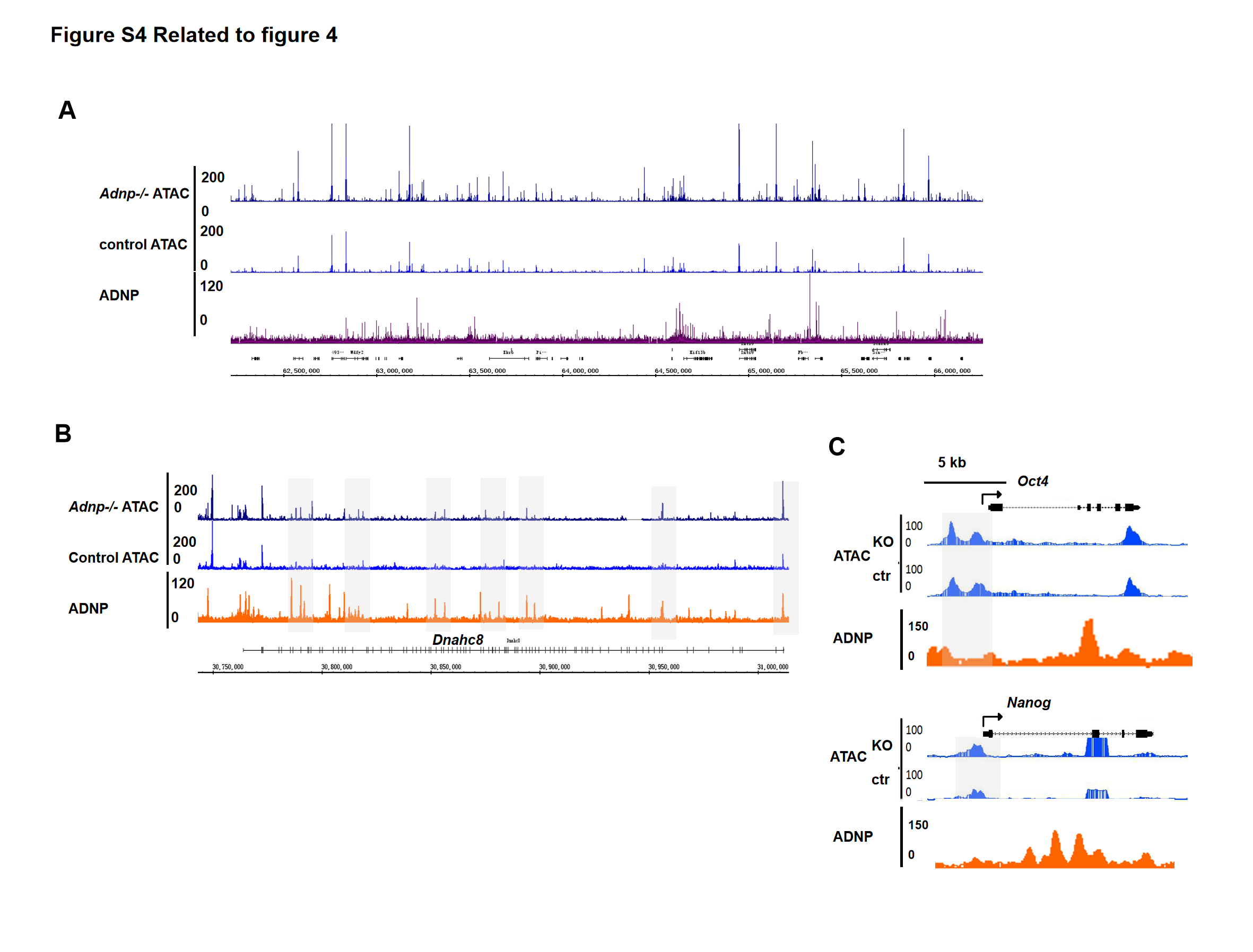

Supplement: FIGURE S4 — (A) ChIP-seq genome browser view of ADNP ChIP-seq and ATAC-seq signals at part of chromosome 5 in control and Adnp-/- ESCs. Note the widespread increased ATAC signals in the absence of ADNP. (B) A example of ChIP-seq genome browser view of ADNP ChIP-seq and ATAC-seq signal at the Dnahc8 locus. Gray: ATAC hypersensitive signal peaks were co-localized with ADNP ChIP-seq peaks. (C) Nucleosome configuration at promoter of the indicated pluripotency genes was not significantly altered in the absence of ADNP. [file Image_4.TIF]

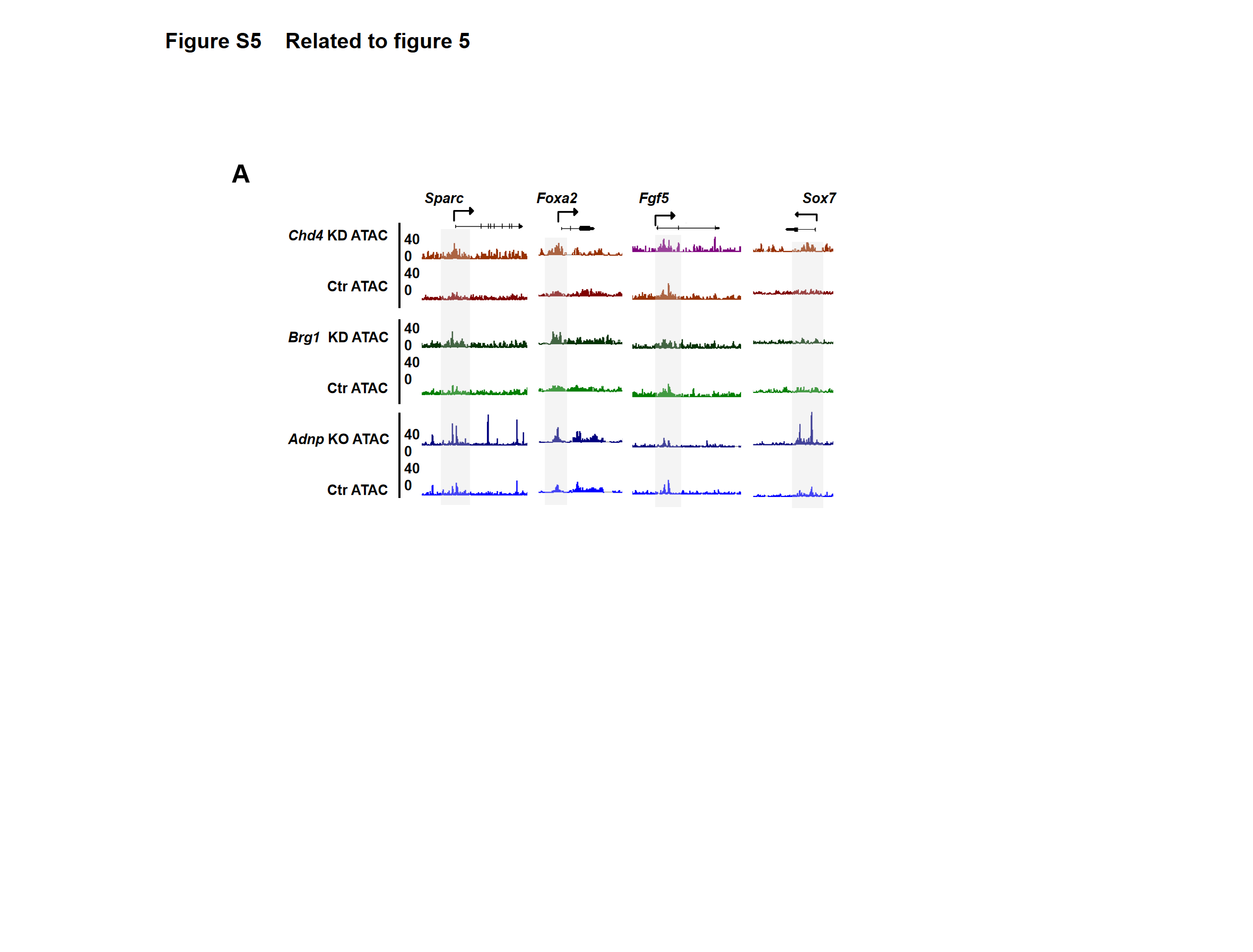

Supplement: FIGURE S5 — (A) A snapshot of ATAC-seq signal change at the indicating loci in the absence of each factor. ATAC-seq experiments were repeated two times for control and Adnp-/- ESCs. ATAC-Seq data for BRG1 and CHD4 were downloaded as described in the text. [file Image_5.TIF]

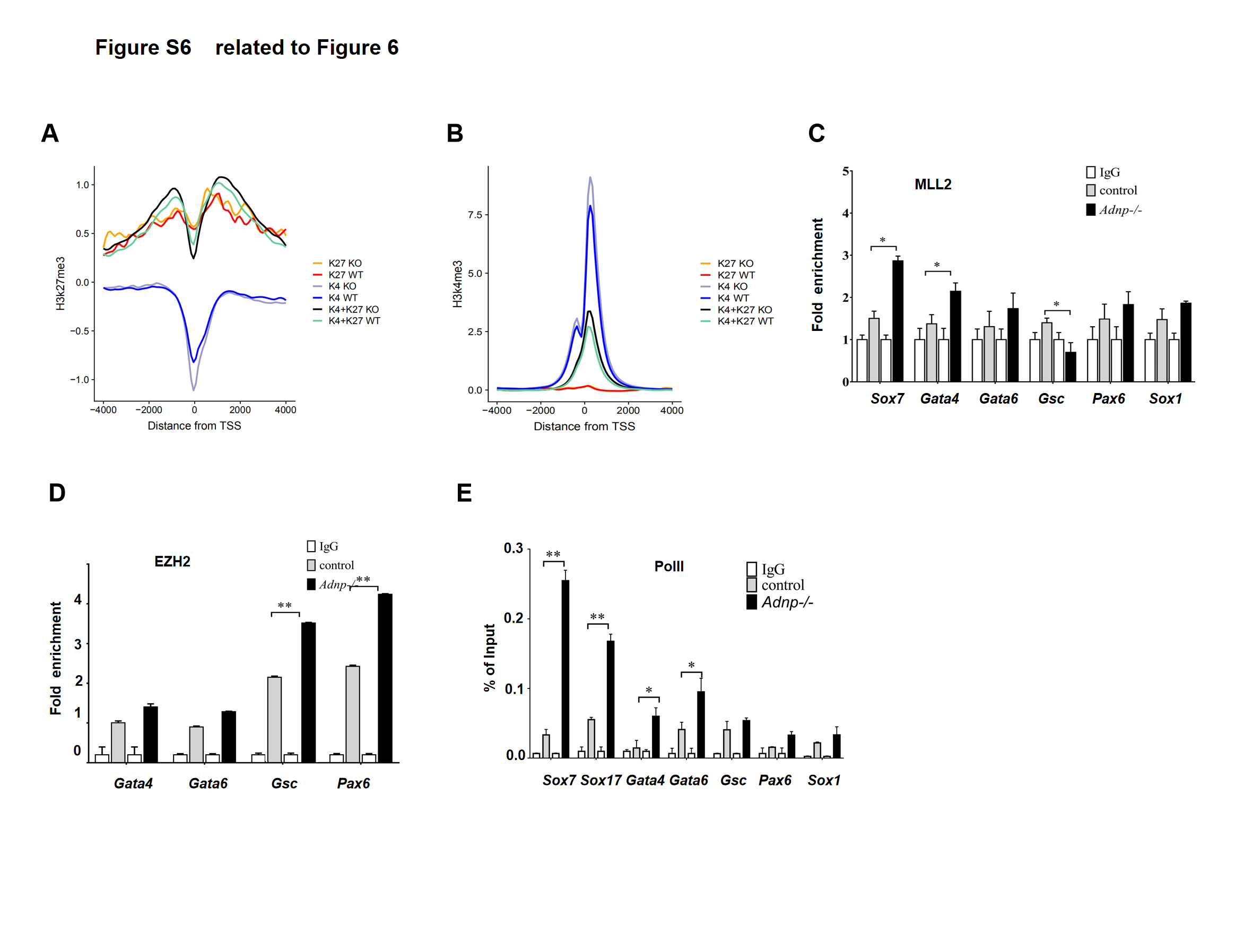

Supplement: FIGURE S6 — (A) A metaplot analysis of H3K4me3 occupancy at TSS region of H3K4me3 only, H3K27me3 only and bivalent genes in control and Adnp-/- ESCs. (B) A metaplot analysis of H3K27me3 occupancy at TSS region of H3K4me3 only, H3K27me3 only and bivalent genes in control and Adnp-/- ESCs. The results were based on two replicates of H3K4me3 and H3K27me3 CHIP-seq experiments. (C) MLL2 enrichment at the indicated genes by ChIP-PCR assay. (D) EZH2 enrichment at the indicated genes by ChIP-PCR assay. (E) Pol II enrichment at indicated genes by ChIP-PCR analysis. All data were based on three repeat experiments. Differences in means were statistically significant when p < 0.05. Significant levels are: ∗p < 0.05; ∗∗P < 0.01; ∗∗∗p < 0.001. [file Image_6.TIF]

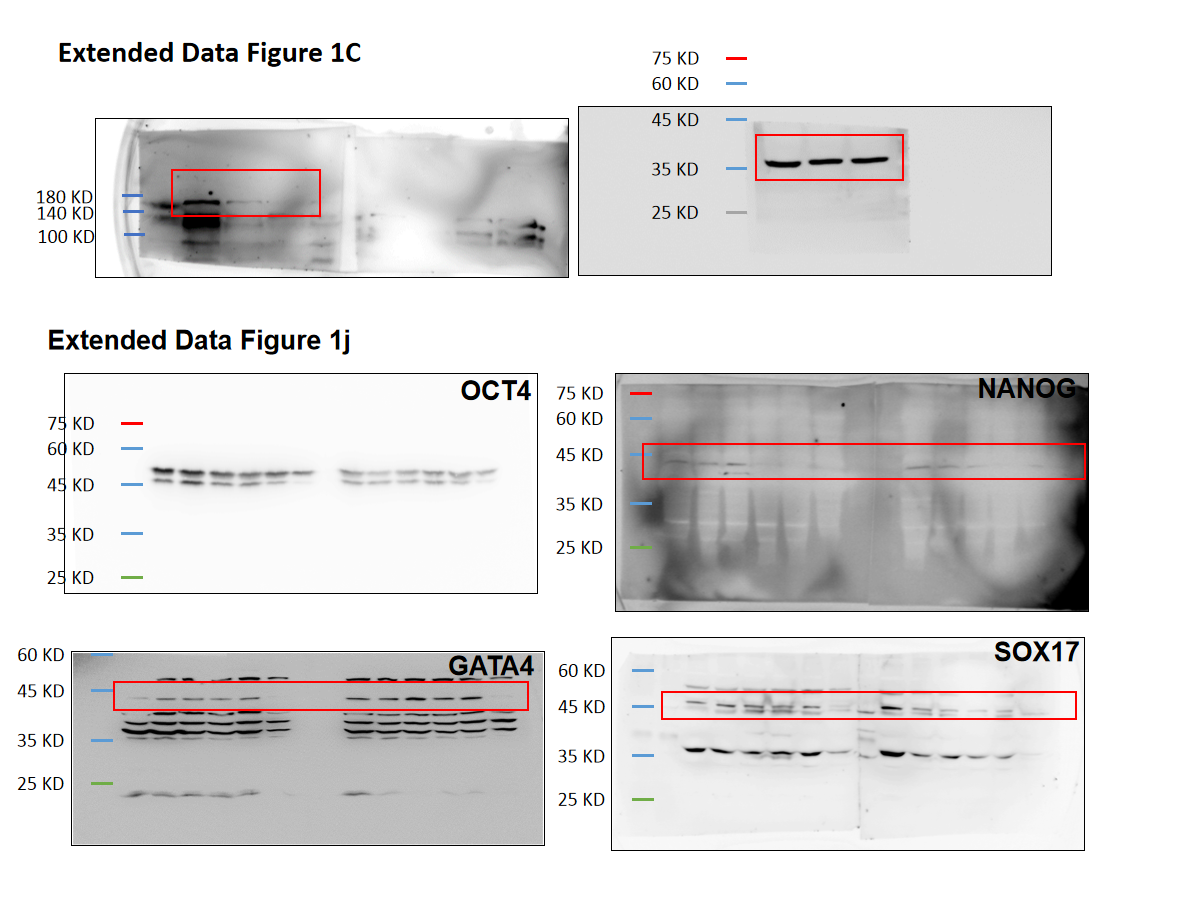

Supplement: Supplementary file 7 [file Image_7.TIF]

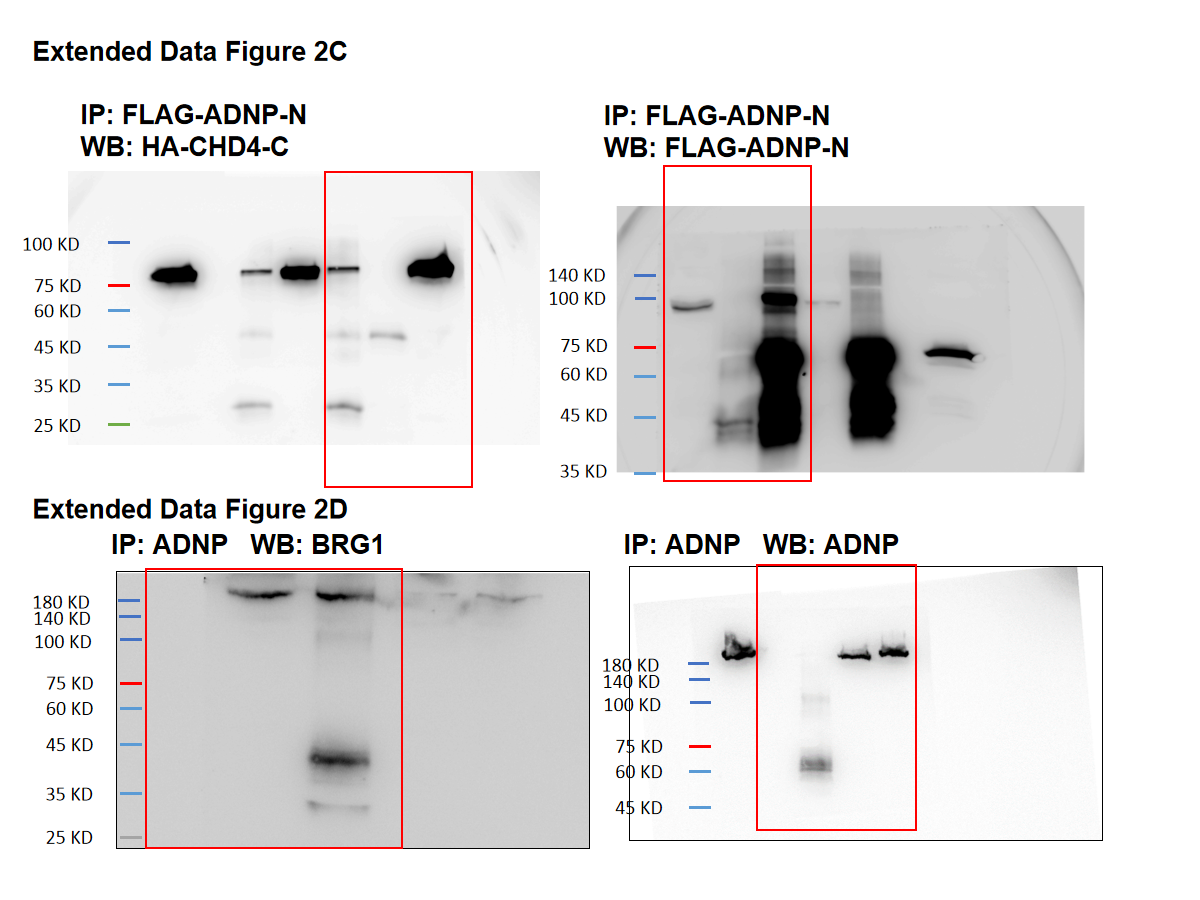

Supplement: Supplementary file 8 [file Image_8.TIF]

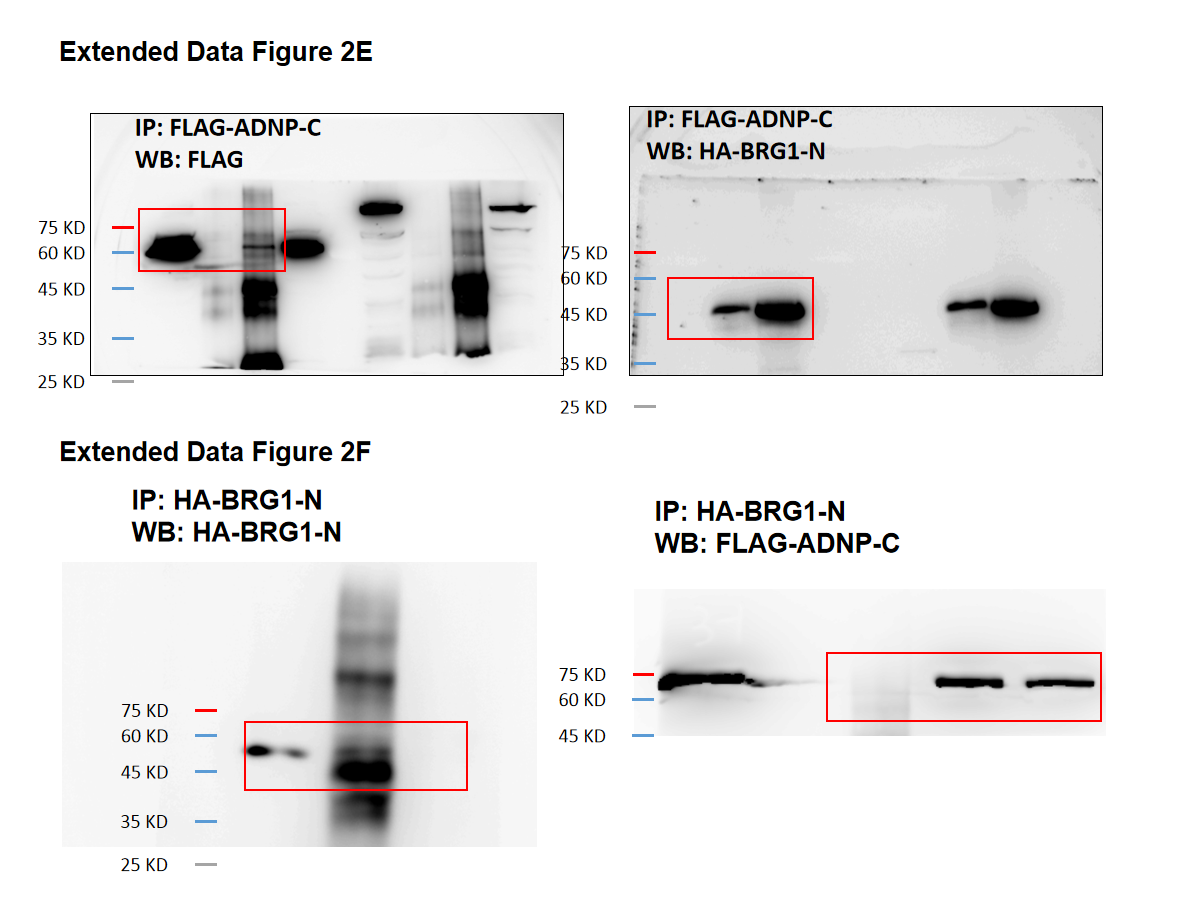

Supplement: Supplementary file 9 [file Image_9.TIF]

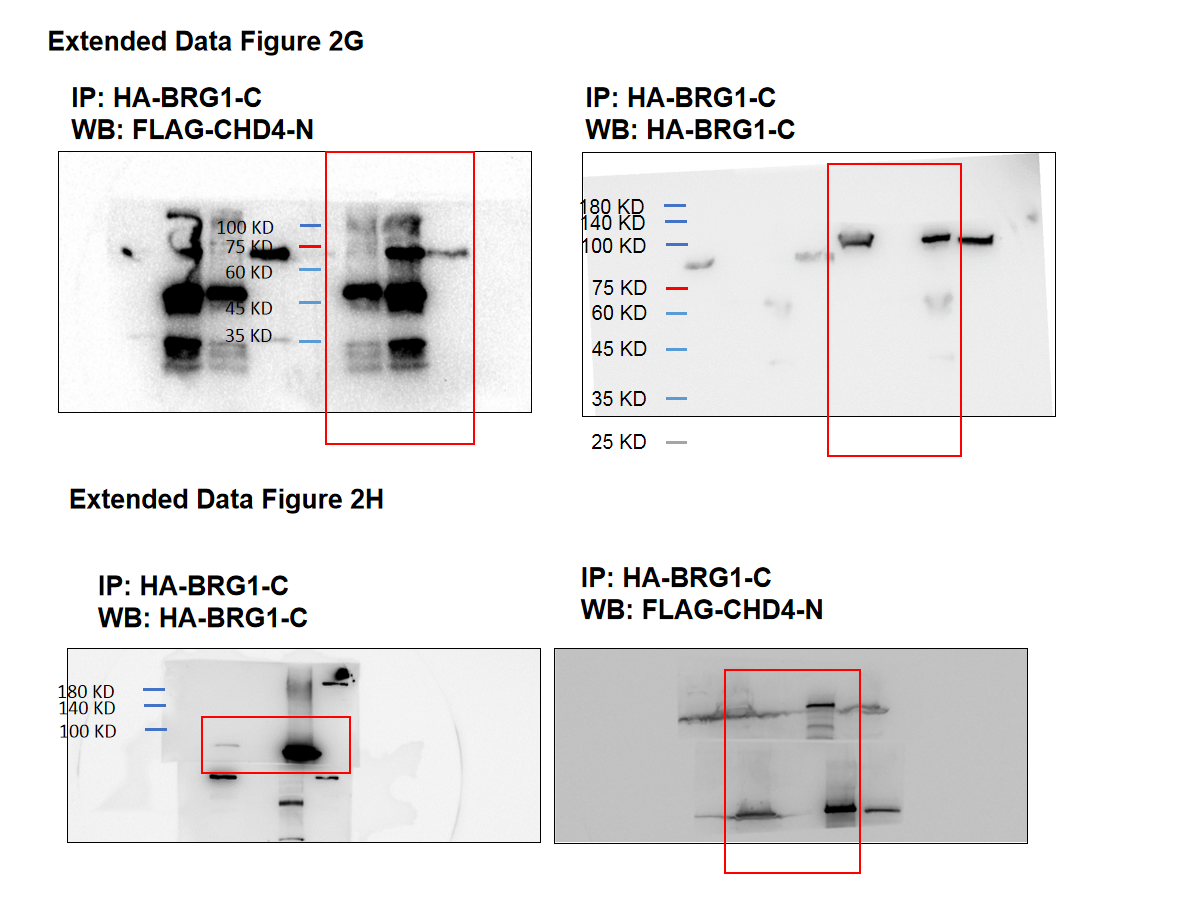

Supplement: Supplementary file 10 [file Image_10.TIF]

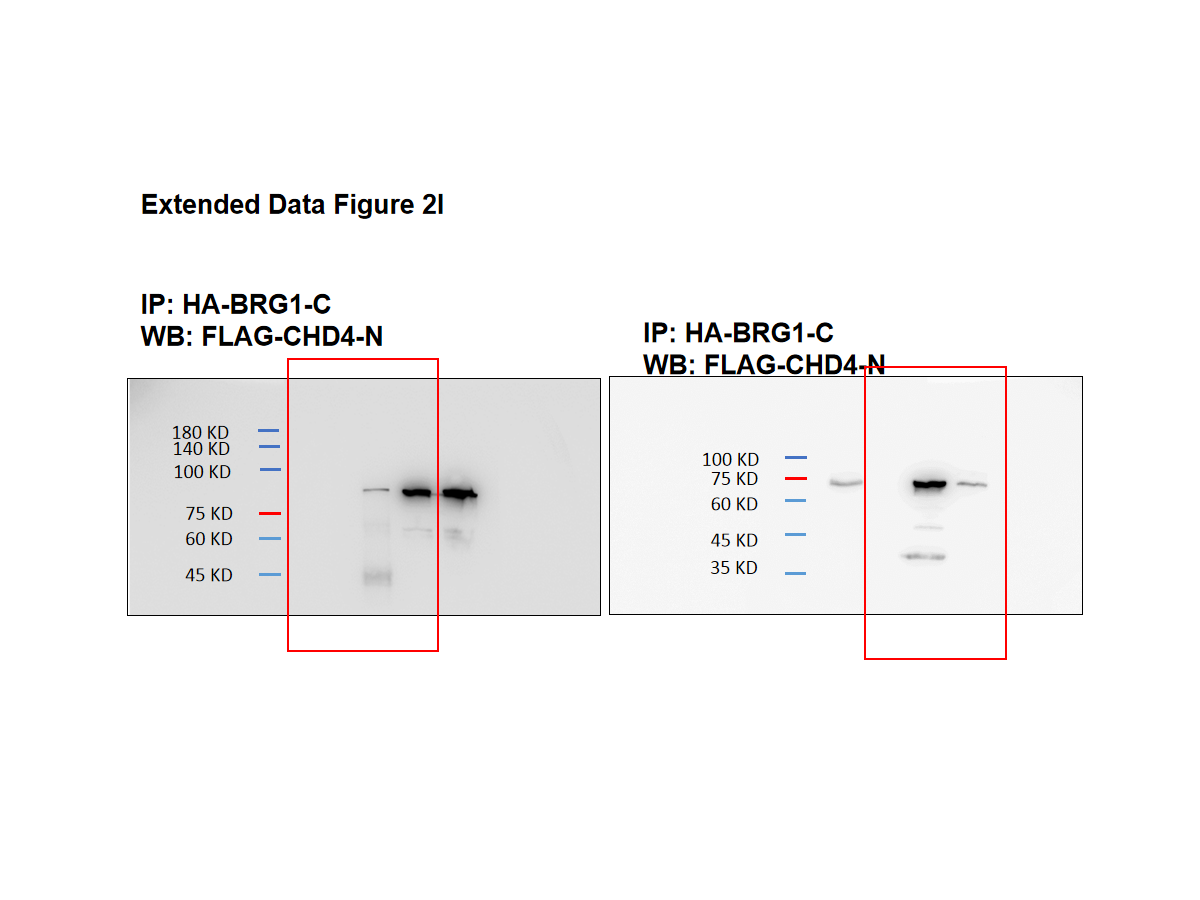

Supplement: Supplementary file 11 [file Image_11.TIF]

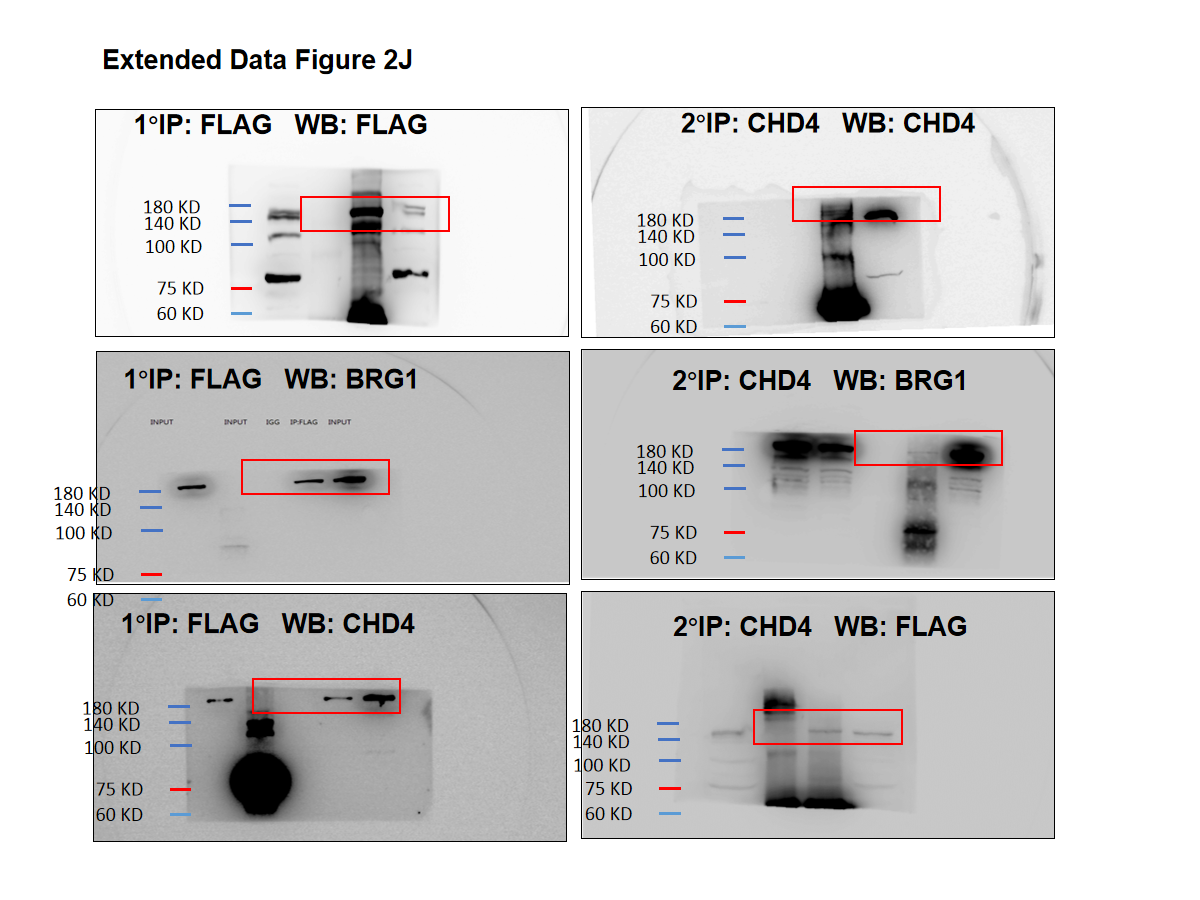

Supplement: Supplementary file 12 [file Image_12.TIF]
